# Synthesis, biological evaluation and docking studies of 1,2,4-oxadiazole linked 5-fluorouracil derivatives as anticancer agents

**DOI:** 10.1186/s13065-021-00757-y

**Published:** 2021-05-04

**Authors:** Ravi Kumar Bommera, Shashikala Kethireddy, Rajeshwar Reddy Govindapur, Laxminarayana Eppakayala

**Affiliations:** 1 Sreenidhi Institute of Science and Technology (Autonomous), Yamnampet, Ghatkesar, Hyderabad, Telangana India; 2 Geethanjali College of Engineering and Technology, (Autonomous), Cheeryal, Keesara, Hyderabad, Telangana India; 3 University of North Carolina Wilmington, Wilmington, NC 28409 USA

**Keywords:** 5-Fluorouracil, Ataluren, Pyrimidine, Oxadiazole and anticancer activity

## Abstract

**Background:**

1,2,4-oxadiazole derivatives exhibited significant anti-cancer activity when they were evaluated, against human cancer cell lines. They also showed anti-inflammatory, analgesic, diabetic, immunosuppressive, α,β_3_-receptor antagonist, antimicrobial, anti-helminthic, histamine-H3 and antiparasitic properties. A pyrimidine analog, 5 fluoro-uracil is a chemotherapeutic drug used for treating multiple solid malignant tumors. But its application is limited, as it has side effects like low bioavailability and high toxicity. Molecular docking is an exemplary tool, helps in identifying target and designing a drug containing high bio-availability and minimum toxicity.

**Results:**

A set of 1,2,4-oxadiazole linked 5-fluoruracil derivatives (7a–j) were synthesized and their structures were confirmed by ^1^HNMR, ^13^CNMR and Mass spectral analysis. Further, these compounds were investigated for their anticancer activity towards a panel of four human cancer cell lines such as (MCF-7, MDA MB-231), lung cancer (A549) and prostate cancer (DU-145) by using MTT method. Among them, compounds 7a, 7b, 7c, 7d and 7i demonstrated more promising anticancer activity than standard.

**Conclusion:**

Synthesized derivatives (7a–j) of 1,2,4-oxadiazole linked 5-fluorouracil and investigated for their anticancer activity towards a panel of four human cancer cell lines.

**Supplementary Information:**

The online version contains supplementary material available at 10.1186/s13065-021-00757-y.

## Background

**Fig. 1:**
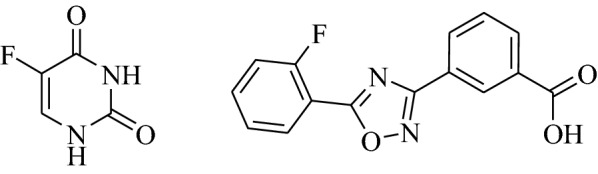
(1) 5-Fluoro uracil, (2) ataluren

Over the past few decades, heterocyclic rings containing nitrogen atoms have played a significant role in medicinal chemistry. They are considered as key templates for the development of new therapeutic agents [1]. Among all the nitrogenated compounds, pyrimidines are a more privileged class of six-membered heterocyclic organic units. They occupy a unique position in medicinal chemistry due to their wide range of biological applications [2–12]. Pyrimidines exist as an essential component in several nucleic acids and therapeutic drugs, such as 5-Fluorouracil (**1**, 5-FU, Fig. 1) [13–16]. The USFDA-approved drug, 5-FU, is one of the most distinguishable chemotherapeutic drugs available. It was first synthesized by Heidelberger and co-workers [17]. It shows antitumor activity by inhibition of thymidylate synthetase enzyme leading to prevention of DNA synthesis [18], and has been used frequently for the treatment of various solid malignant tumors [19–21]. However, it has limited clinical applications because of several side effects, including poor tumor selectivity, toxicity, lower drug-resistance, gastrointestinal toxicity, and adverse effects on central nervous system [22, 23]. Previously, many researchers have developed several 5-FU contained compounds to overcome such side effects [24]. On the other hand, oxadiazoles are a unique class of nitrogen and oxygen atoms containing five-membered ring heterocyclic core units [25]. They are frequently found in marine organisms [26]. These are more attractable heterocyclic structural framework to medicinal chemist [27], due to their broad spectrum of biological properties including (2S)cannabinoid receptor 2 (CB2) [28], immunosuppressive [29], muscarinic [30], α,β_3_-receptor antagonist [31], antimicrobial [32], insecticides [33], histamine-H3 [34], anti-inflammatory [35], analgesic [36], diabetic, anticancer, antiparasitic and anti-helminthic properties. The US FDA approved drug such as ataluren (**2**), contains 1,2,4-oxadiazole framework as a part of the structure and is used for the treatment of muscular dystrophy [37–39].

The above biological findings and the continuous of demand for the new anticancer agents prompted us to design and synthesize a set of 1,2,4-oxadiazole linked 5-fluorouracil derivatives (**7a**–**j**). Their structures were confirmed by ^1^HNMR, ^13^CNMR and mass spectral data. Their anticancer activity towards four human cancer cells such as breast cancer (MCF-7, MDA MB-231), lung cancer (A549) and prostate cancer (DU-145) were evaluated.

## Results and discussion

**Scheme 1:**
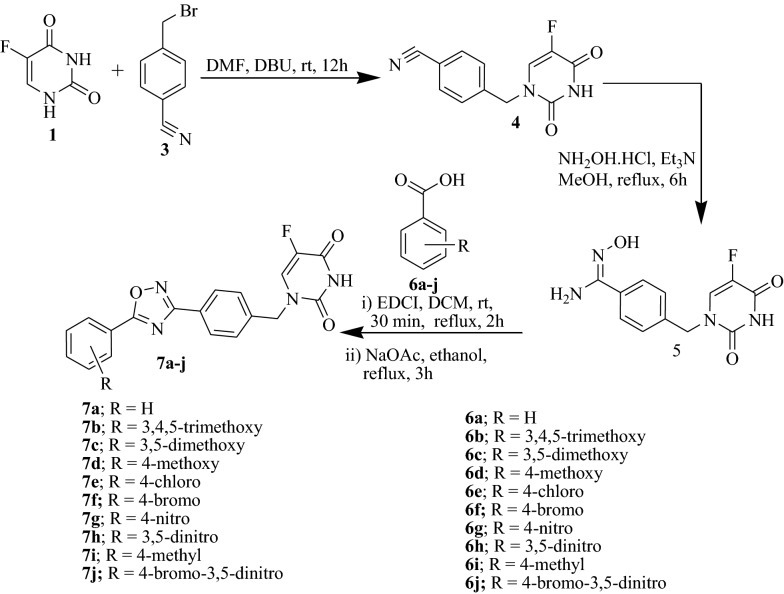
Synthesis of oxadiazoles

The synthesis of 1,2,4-oxadiazole linked 5-fluorouracil derivatives (**7a**–**j**) described in this study are outlined in Scheme 1. Commercially available 5-fluorouracil (**1**) was treated with 4-(bromomethyl)benzonitrile (**3**) in the presence of a base, DBU and anhydrous DMF at room temperature for 12 h to give an intermediate compound **4** with 67% yield. The resulting nitrile intermediate **4** was reacted with hydroxylamine hydrochloride in triethyl amine base in methanol at reflux for 6 h to afford pure amidoxime compound **5** with 82% yield. Further, intermediate **5** was subjected to cyclization with substituted aromatic carboylic acids (**6a**–**j**) in presence of a coupling reagent, EDC·HCl and a base sodium acetate in ethanol at reflux for 3 h to afford pure final compounds **7a**–**j** in good yields.

## Experimental section

### Materials and methods

All chemicals and reagents were obtained from Aldrich (Sigma-Aldrich, St. Louis, MO, USA), Lancaster (Alfa Aesar, Johnson Matthey Company, Ward Hill, MA, USA) and were used without further purification. Human cancer cell lines such as (MCF-7, MDA MB-231 were collected from Sigma-Aldrich. Reactions were monitored by TLC, performed on silica gel glass plates containing 60 F-254, and visualization on TLC was achieved by UV light or iodine indicator. ^1^H and ^13^C NMR spectra were recorded on Gemini Varian-VXR-unity (400 MHz, 300 MHz) instrument. Chemical shifts (d) are reported in ppm downfield from internal TMS standard. ESI spectra were recorded on Micro mass, Quattro LC using ESI+ software with capillary voltage 3.98 kV and ESI mode positive ion trap detector. Melting points were determined with an electro thermal melting point apparatus and are uncorrected.

### 4-((5-Fluoro-3,4-dihydro-2,4-dioxopyrimidin-1(2H)-yl)methyl)benzonitrile (4)

Compound **3** (14.9 g, 0.076 mol) was dissolved in 10 ml of dry DMF and added dropwise to a solution of 5-fluorouracil (**1**) (10 g, 0.076 mol) and DBU (12.6 ml, 0.084 mol) in dry DMF (40 ml) under N_2_ atmosphere at 4 °C. The reaction mixture was allowed to stir at room temperature overnight, and then concentrated. The crude product was purified using flash chromatography (hexane/ethyl acetate = 1:1) to afford corresponding product **4** as 12.6 g with 67% yield. IR (KBr): 3265 (N–H stretching vibration), 3021 (Ar–C–H str.), 2893 (C–H str.), 1632 (NH(C=O)–C=C– stretching vibration of amide), 1562, 1469 (C=N and C=C stretching vibration of pyridine Nucleus), 988 (C–F stretching vibration), 782 (2-Substituted pyridine),^1^H-NMR (300 MHz, DMSO-d6): $\updelta$ 4.86 (s, 2H), 7.34 (d, 2H, J = 8.0 Hz), 7.72 (d, 2H, J = 8.0 Hz), 8.23 (d, 1H, J = 6.1 Hz), 11.89 (s, 1H); ^13^C NMR (75 MHz, DMSO-d6): $\delta$ 156.8, 149.3, 141.6, 141.0, 134.3, 130.1, 126.1, 118.3, 109.1, 49.7; MS (ESI): m/z 246 [M+H]^+^.

### (1E)-4-((5-Fluoro-3,4-dihydro-2,4-dioxopyrimidin-1(2H)-yl)methyl)-N'-hydroxybenzamidine (5)

To a solution of compound 4-((5-fluoro-3,4-dihydro-2,4-dioxopyrimidin-1(2H)-yl) methyl)benzonitrile (**4**) (12 g, 0.049 mol) in methanol was added Et_3_N (20 ml, 0.146 mol) and NH_2_OH·HCl (10.2 g, 0.146 mol), and the reaction mixture was refluxed for 6 h. The reaction mixture was concentrated under vacuum; the residue was diluted with water and extracted with ethyl acetate. The combined organic layers were dried over anhydrous Na_2_SO_4_, concentrated in vacuo and purified by flash column chromatography on silica gel by using ethyl acetate/hexane (9:1) to afford the pure product **5** as 11.2 g with 82% yield. IR (KBr): 3314 (N–H stretching vibration), 3052 (Ar–C–H str.), 2962 (C–H str.), 1684 (NH(C=O)–C=C– stretching vibration of amide), 1684, 1485 (C=N and C=C stretching vibration of pyridine Nucleus), 974 (C–F stretching vibration), 766 (2-Substituted pyridine), ^1^H NMR (300 MHz, DMSO-d6): $\delta$ 4.82 (s, 2H), 5.88 (s, 2H), 7.26 (d, 2H, J = 8.0 Hz), 7.70 (d, 2H, J = 8.0 Hz), 8.22 (d, 1H, J = 6.0 Hz), 11.89 (s, 1H), 14.35 (s, 1H); ^13^C NMR (75 MHz, DMSO-d6): $\delta$ 179.6*,* 156.7, 150.3, 141.3, 140.7, 134.1, 131.0, 129.3, 126.2, 49.2; MS (ESI): m/z 279 [M+H]^+^.

### 1-(4-(5-Phenyl-1,2,4-oxadiazol-3-yl)benzyl)-5-fluoropyrimidine-2,4(1H,3H)-dione (7a)

A solution of substituted benzoic acid (**6a**) (0.16 ml, 0.0017 mol) in anhydrous CH_2_Cl_2_ (50 ml) was cooled to 0 °C and ethyl-(*N*ʹ,*N*ʹ-dimethylamino)propylcarbodiimide hydrochloride (EDC·HCl) (418 mg, 0.00269 mol) was added to it under nitrogen atmosphere. The reaction mixture was stirred at 0 °C for an additional half an hour. To this mixture, (1E)-4-((5-fluoro-3,4-dihydro-2,4-dioxopyrimidin-1(2H)-yl)methyl)-*N*ʹ-hydroxybenzamidine (**5**) (500 mg, 0.0017 mmol) was added and stirring was continued for another half an hour at 0 °C. The reaction mixture was slowly allowed to attain room temperature with continuous stirring. Then it was heated to 110 °C for 2 h. The progress of the reaction was monitored by TLC. After completion of the reaction, the reaction mixture was cooled down to the room temperature, to obtain the solid product, which was then filtered and washed with CH_2_Cl_2_. The resultant white solid was heated for 3 h by dissolving it in a mixture of ethanol (20 ml), sodium acetate (83 mg, 0.0017 mmol) and water (3 ml). The reaction mixture was then cooled down to room temperature. The resultant solid was filtered and purified by recrystallization using ethanol to obtain the desired solid compound **7a** with 32% (210.4 mg) yield.

Mp: 316–318 °C, IR (KBr): 3176 (N–H stretching vibration), 2965 (Ar–C–H str.), 2944 (C–H str.), 1642 (NH(C=O)–C=C– stretching vibration of amide), 1598, 1425 (C=N and C=C stretching vibration of pyridine Nucleus), 1065 (C–F stretching vibration), 776 (2-Substituted pyridine), ^1^H NMR (300 MHz, DMSO-d6): $\delta$ 4.98 (s, 2H), 7.34–7.45 (m, 3H), 7.61 (d, 2H, J = 8.1 Hz), 7.94 (d, 2H, J = 7.6 Hz), 8.24 (d, 1H, J = 6.1 Hz), 8.28 (d, 2H, J = 8.1 Hz), 11.88 (s, 1H); ^13^C NMR (75 MHz, DMSO-d6): $\delta$ 52.8, 127.5, 128.4, 128.7, 129.5, 130.4, 131.5, 131.8, 134.2, 134.7, 138.4, 140.3, 145.5, 151.7, 155.6, 157.8, 158.3, 163.7; MS (ESI): m/z 365 [M+H]^+^.

### 1-(4-(5-(3,4,5-trimethoxyphenyl)-1,2,4-oxadiazol-3-yl)benzyl)-5-fluoropyrimidine-2,4(1H,3H)-dione (7b)

The compound, **7b** was prepared following the method described for the preparation of the compound **7a**, employing **5** (500 mg, 0.0017 mol) with 3,4,5-trimethoxybenzoic acid (**6b**) (361 mg, 0.0017 mol), EDC·HCl (418 mg, 0.00269 mol) and sodium acetate (83 mg, 0.0017 mmol) to afford pure compound **7b**, 308.4 mg in 38% yield. Mp: 330–332 °C,IR (KBr): 3181 (N–H stretching vibration), 3062 (Ar–C–H str.), 2923 (C–H str.), 1657 (NH(C=O)–C=C– stretching vibration of amide), 1602, 1403 (C=N and C=C stretching vibration of pyridine Nucleus), 1093 (C–F stretching vibration), 773 (2-Substituted pyridine), ^1^HNMR (300 MHz, DMSO-d6): $\delta$ 3.88 (s, 3H), 3.91 (s, 6H), 4.90 (s, 2H), 7.63 (d, 2H, J = 8.0 Hz), 7.69 (s, 2H), 8.23 (d, 1H, J = 6.1 Hz), 8.28 (d, 2H, J = 8.0 Hz), 11.87 (s, 1H); ^13^CNMR (75 MHz, DMSO-d6): $\delta$ 52.7, 57.6, 61.5, 106.4, 12.6, 128.5, 128.7, 129.4, 130.4, 131.5, 134.7, 138.2, 140.2, 143.5, 145.4, 151.7, 155.6, 155.8, 157.6, 158.4, 165.7; MS (ESI): m/z 455 [M+H]^+^.

### 1-(4-(5-(3,5-dimethoxyphenyl)-1,2,4-oxadiazol-3-yl)benzyl)-5-fluoropyrimidine-2,4(1H,3H)-dione (7c)

The compound, **7c** was prepared similar to the preparation of the compound **7a**, in which compound **5** (500 mg, 0.0017 mol) was treated with 3,5-dimethoxybenzoic acid (**6c**) (309 mg, 0.0017 mol), EDC·HCl (418 mg, 0.00269 mol) and sodium acetate (83 mg 0.0017 mol) to afford pure compound **7c**, 284.2 mg in 37% yield. Mp: 345–347 °C, IR (KBr): 3181 (N–H stretching vibration), 3065 (Ar–C–H str.), 2912 (C–H str.), 1621 (NH(C=O)–C=C– stretching vibration of amide), 1612, 1423 (C=N and C=C stretching vibration of pyridine Nucleus), 1053 (C–F stretching vibration), 762 (2-Substituted pyridine), ^1^H NMR (300 MHz, DMSO-d6): $\delta$ 3.79 (s, 6H), 4.98 (s, 2H), 6.98 (s, 1H), 7.61 (d, 2H, J = 8.1 Hz), 7.67 (s, 2H), 8.24 (d, 1H, J = 6.1 Hz), 8.29 (d, 2H, J = 8.1 Hz), 11.89 (s, 1H); ^13^C NMR (75 MHz, DMSO-d6): $\delta$ 52.6, 57.6, 104.6, 105.7, 127.6, 128.4, 128.7, 130.4, 134.5, 134.8, 138.5, 140.2, 145.8, 151.7, 155.9, 157.4, 158.7, 162.6, 165.7; MS (ESI): m/z 425 [M+H]^+^.

### 1-(4-(5-(4-Methoxyphenyl)-1,2,4-oxadiazol-3-yl)benzyl)-5-fluoropyrimidine-2,4(1H,3H)-dione (7d)

The compound, **7d** was prepared following the method described for the preparation of the compound **7a**, employing **5** (500 mg, 0.0017 mol) with 4-methoxybenzoic acid (**6d**) (258 mg, 0.0017 mol), EDC·HCl (418 mg, 0.00269 mol) and sodium acetate (83 mg, 0.0017 mol) to afford pure compound **7d**, 291.3 mg in 41% yield. Mp: 342–344 °C, IR (KBr): 3181 (N–H stretching vibration), 3021 (Ar–C–H str.), 2895 (C–H str.), 1641 (NH(C=O)–C=C– stretching vibration of amide), 1618, 1386 (C=N and C=C stretching vibration of pyridine Nucleus), 1093 (C–F stretching vibration), 784 (2-Substituted pyridine), ^1^H NMR (300 MHz, DMSO-d6): $\delta$ 3.96 (s, 3H), 5.06 (s, 2H), 7.17 (d, 2H, J = 7.7 Hz), 7.63 (d, 2H, J = 8.1 Hz), 8.09 (d, 2H, J = 7.7 Hz), 8.24 (d, 1H, J = 6.1 Hz), 8.28 (d, 2H, J = 8.1 Hz), 11.89 (s, 1H); ^13^C NMR (75 MHz, DMSO-d6): $\delta$ 52.8, 57.8, 118.4, 118.9, 127.4, 128.6, 128.9, 130.5, 134.5, 138.2, 140.6, 145.6, 151.8, 155.6, 157.3, 158.4, 162.5, 165.8; MS (ESI): m/z 395 [M+H]^+^.

### 1-(4-(5-(4-Chlorophenyl)-1,2,4-oxadiazol-3-yl)benzyl)-5-fluoropyrimidine-2,4(1H,3H)-dione (7e)

This compound, **7e** was prepared following similar method used for the preparation of the compound **7a**, employing **5** (500 mg, 0.0017 mol) with 4-chlorobenzoic acid (**6e**) (266 mg, 0.0017 mol), EDC·HCl (418 mg, 0.00269 mol) and sodium acetate (83 mg, 0.0017 mol) to afford pure compound **7e**, 313.5 mg in 44% yield. Mp: 338–340 °C,IR (KBr): 3163 (N–H stretching vibration), 3075 (Ar–C–H str.), 2897 (C–H str.), 1691 (NH(C=O)–C=C– stretching vibration of amide), 1610, 1422 (C=N and C=C stretching vibration of pyridine Nucleus), 1094 (C–F stretching vibration), 794 (2-Substituted pyridine), ^1^H NMR (300 MHz, DMSO-d6): $\delta$ 5.19 (s, 2H), 7.61 (d, 2H, J = 8.1 Hz), 7.72 (d, 2H, J = 7.8 Hz), 8.12 (d, 2H, J = 7.8 Hz), 8.25 (d, 1H, J = 6.2 Hz), 8.30 (d, 2H, J = 8.1 Hz), 11.89 (s, 1H); ^13^C NMR (75 MHz, DMSO-d6): $\delta$ 53.2, 127.6, 128.7, 128.9, 129.5, 130.4, 130.8, 133.4, 134.5, 134.7, 138.5, 140.3, 145.6, 151.7, 155.6, 157.4, 158.7, 165.8; MS (ESI): m/z 399 [M+H]^+^.

### 1-(4-(5-(4-Bromophenyl)-1,2,4-oxadiazol-3-yl)benzyl)-5-fluoropyrimidine-2,4(1H,3H)-dione (7f)

The compound **7f** was prepared using the method described for the preparation of the compound **7a**, employing **5** (500 mg, 0.0017 mol) with 4-bromobenzoic acid (**6f**) (341 mg, 0.0017 mol), EDC·HCl (418 mg, 0.00269 mol) and sodium acetate (83 mg, 0.0017 mol) to afford pure compound **7f**, 372.4 mg in 47% yield. Mp: 362–364 °C, IR (KBr): 3203 (N–H stretching vibration), 3055 (Ar–C–H str.), 2965 (C–H str.), 1643 (NH(C=O)–C=C– stretching vibration of amide), 1632, 1403 (C=N and C=C stretching vibration of pyridine Nucleus), 1093 (C–F stretching vibration), 769 (2-Substituted pyridine), ^1^H NMR (300 MHz, DMSO-d6): $\delta$ 5.17 (s, 2H), 7.64 (d, 2H, J = 8.1 Hz), 7.76–7.88 (m, 4H), 8.23 (d, 1H, J = 6.2 Hz), 8.31 (d, 2H, J = 8.1 Hz), 11.89 (s, 1H); ^13^C NMR (75 MHz, DMSO-d6): $\delta$ 52.9, 126.7, 127.6, 128.7, 128.9, 129.2, 130.6, 133.4, 134.5, 134.8, 138.6, 140.6, 145.8, 151.6, 155.6, 157.8, 158.9, 165.7; MS (ESI): m/z 445 [M+H]^+^.

### 1-(4-(5-(4-Nitrophenyl)-1,2,4-oxadiazol-3-yl)benzyl)-5-fluoropyrimidine-2,4(1H,3H)-dione (7g)

The compound, **7g** was prepared as described above, employing **5** (500 mg, 0.0017 mol) with 4-nitrobenzoic acid (**6g**) (284 mg, 0.0017 mol), EDC·HCl (418 mg, 0.00269 mol) and sodium acetate (83 mg, 0.0017 mol) to afford pure compound **7g**, 367.3 mg in 50% yield. Mp: 370–372 °C, IR (KBr): 3176 (N–H stretching vibration), 2906 (Ar–C–H str.), 2966 (C–H str.), 1668 (NH(C=O)–C=C– stretching vibration of amide), 1631, 1396 (C=N and C=C stretching vibration of pyridine Nucleus), 1112 (C–F stretching vibration), 791 (2-Substituted pyridine), ^1^H NMR (300 MHz, DMSO-d6): $\delta$ 5.28 (s, 2H), 7.65 (d, 2H, J = 8.2 Hz), 7.96 (d, 2H, J = 8.1 Hz), 8.14 (d, 2H, J = 8.1 Hz), 8.24 (d, 1H, J = 6.2 Hz), 8.33 (d, 2H, J = 8.2 Hz), 11.91 (s, 1H); ^13^C NMR (75 MHz, DMSO-d6): $\delta$ 53.5, 126.7, 127.3, 127.8, 128.4, 128.7, 130.5, 134.7, 138.4, 139.6, 140.6, 145.8, 149.4, 151.7, 155.6, 157.3, 158.4, 165.8; MS (ESI): m/z 410 [M+H]^+^.

### 1-(4-(5-(3,5-Dinitrophenyl)-1,2,4-oxadiazol-3-yl)benzyl)-5-fluoropyrimidine-2,4(1H,3H)-dione (7h)

The compound **7h** was prepared as per the method used for the preparation of the compound **7a**, in which compound **5** (500 mg, 0.0017 mol) treated with 3,5-dinitrobenzoic acid (**6h**) (360 mg, 0.0017 mol), EDC·HCl (418 mg, 0.00269 mol) and sodium acetate (83 mg, 0.0017 mol) to afford pure compound **7h**, 342.8 mg in 42% yield. Mp: 376–378 °C, IR (KBr): 3206 (N–H stretching vibration), 3112 (Ar–C–H str.), 2887 (C–H str.), 1648 (NH(C=O)–C=C– stretching vibration of amide), 1603, 1403 (C=N and C=C stretching vibration of pyridine Nucleus), 1114 (C–F stretching vibration), 769 (2-Substituted pyridine), ^1^H NMR (300 MHz, DMSO-d6): $\delta$ 5.37 (s, 2H), 7.66 (d, 2H, J = 8.3 Hz), 8.25 (d, 1H, J = 6.3 Hz), 8.37 (d, 2H, J = 8.3 Hz), 8.52 (s, 2H), 8.89 (s, 1H), 11.91 (s, 1H); ^13^C NMR (75 MHz, DMSO-d6): $\delta$ 53.7, 121.6, 127.6, 128.5, 128.9, 129.3, 130.7, 134.5, 138.6, 138.9, 140.4, 145.8, 149.7, 151.7, 155.8, 157.7, 158.7, 165.9; MS (ESI): m/z 455 [M+H]^+^.

### 1-(4-(5-p-Tolyl-1,2,4-oxadiazol-3-yl)benzyl)-5-fluoropyrimidine-2,4(1H,3H)-dione (7i)

The compound **7i** was prepared as described above. In this, compound **5** (500 mg, 0.0017 mol) was reacted with 4-methylbenzoic acid (**6i**) (231 mg, 0.0017 mmol), EDC·HCl (418 mg, 0.00269 mol) and sodium acetate (83 mg, 0.0017 mol) to afford pure compound **7i**, 239.6 mg in 35% yield. Mp: 356–358 °C, IR (KBr): 3224 (N–H stretching vibration), 3102 (Ar–C–H str.), 2986 (C–H str.), 1643 (NH(C=O)–C=C– stretching vibration of amide), 1632, 1405 (C=N and C=C stretching vibration of pyridine Nucleus), 1087 (C–F stretching vibration), 756 (2-Substituted pyridine), ^1^H NMR (300 MHz, DMSO-d6): $\delta$ 2.69 (s, 3H), 5.10 (s, 2H), 7.37 (d, 2H, J = 7.8 Hz), 7.62 (d, 2H, J = 8.1 Hz), 7.86 (d, 2H, J = 7.8 Hz), 8.23 (d, 1H, J = 6.1 Hz), 8.31 (d, 2H, J = 8.1 Hz), 11.89 (s, 1H); ^13^C NMR (75 MHz, DMSO-d6): $\delta$ 35.8, 52.7, 127.5, 128.7, 128.9, 129.5, 130.5, 131.7, 133.4, 134.8, 138.6, 140.6, 141.8, 145.7, 151.7, 157.8, 158.9, 165.6; MS (ESI): m/z 379 [M+H]^+^.

### 1-(4-(5-(4-Bromo-3,5-dinitrophenyl)-1,2,4-oxadiazol-3-yl)benzyl)-5-fluoropyrimidine-2,4(1H,3H)-dione (7j)

This compound **7j** was prepared following the method described for the preparation of the compound **7a**, in which compound **5** (500 mg, 0.0017 mol) was made to react with 4-bromo-3,5-dinitrobenzoic acid (**6j**) (494 mg, 0.0017 mol), EDC·HCl (418 mg, 0.00269 mol) and sodium acetate (83 mg, 0.0017 mol) to afford pure compound **7j**, 356.9 mg in 37% yield. Mp: 386–388 °C, IR (KBr): 3125 (N–H stretching vibration), 3098 (Ar–C–H str.), 2995 (C–H str.), 1664 (NH(C=O)–C=C– stretching vibration of amide), 1609, 1409 (C=N and C=C stretching vibration of pyridine Nucleus), 1076 (C–F stretching vibration), 748 (2-Substituted pyridine), ^1^H NMR (300 MHz, DMSO-d6): $\delta$ 5.40 (s, 2H), 7.65 (d, 2H, J = 8.3 Hz), 8.25 (d, 1H, J = 6.3 Hz), 8.37 (d, 2H, J = 8.3 Hz), 8.68 (s, 2H), 11.92 (s, 1H); ^13^C NMR (75 MHz, DMSO-d6): $\delta$ 53.7, 122.7, 27.6, 128.5, 128.9, 130.6, 132.6, 134.8, 136.7, 138.4, 140.6, 145.8, 151.7, 157.6, 158.9, 166.8; MS (ESI): m/z 534 [M+H]^+^.

## Biological evaluation

### In vitro cytotoxicity

The target compounds (**7a**–**j**) were examined for their anticancer activity against a panel of four human cancer cell lines including breast cancer (MCF-7, MDA MB-231), lung cancer (A549) and prostate cancer (DU-145) by using MTT method. Etoposide was used as reference standard and the obtained results were summarized in Table 1.

**Table 1:** Anticancer activity of newly synthesized compounds **7a**–**j** with IC_50_ in µM

Compound	MCF-7	A549	DU-145	MDA MB-231
**7a**	0.76 ± 0.044	0.18 ± 0.019	1.13 ± 0.55	0.93 ± 0.013
**7b**	0.011 ± 0.009	0.053 ± 0.0071	0.017 ± 0.0062	0.021 ± 0.0028
**7c**	0.88 ± 0.073	1.44 ± 0.32	1.28 ± 0.27	1.95 ± 0.19
**7d**	1.78 ± 0.22	1.67 ± 0.49	2.10 ± 1.09	2.34 ± 1.10
**7e**	3.45 ± 1.87	6.34 ± 3.24	ND	3.98 ± 1.88
**7f**	5.98 ± 2.56	ND	6.22 ± 2.91	ND
**7g**	9.22 ± 5.66	10.5 ± 5.72	4.33 ± 4.25	2.75 ± 1.24
**7h**	8.21 ± 5.19	11.3 ± 6.32	ND	ND
**7i**	2.17 ± 1.66	1.88 ± 0.25	2.65 ± 1.26	2.14 ± 0.94
**7j**	7.12 ± 4.30	13.6 ± 7.56	ND	19.4 ± 8.11
**Etoposide**	2.11 ± 0.024	3.08 ± 0.135	1.97 ± 0.45	1.91 ± 0.84

*ND* not determined

Among the compounds (**7a**–**j**) synthesized **7a**, **7b**, **7c**, **7d** and **7i** possessed good activity with IC_50_ values ranging from 0.011 ± 0.009 to 19.4 ± 8.11 µM and standard reference showed IC_50_ values range from 1.91 ± 0.84 to 3.08 ± 0.135 µM, respectively. The structure–activity relationship (SAR) studies indicated that the compound **7a** without substituent on the phenyl ring attached to 1,2,4-oxadiazole moiety has showed good anticancer activity against four cell lines (MCF-7 = 0.76 ± 0.044 µM; A549 = 0.18 ± 0.019 µM; DU145 = 1.13 ± 0.55 µM and MDA MB-231 = 0.93 ± 0.013 µM). Up on introduction of electron-donating 3,4,5-trimethoxy group on the phenyl ring resulted compound **7b**, showed more significant anticancer activity (MCF-7 = 0.011 ± 0.009 µM; A549 = 0.053 ± 0.0071 µM; DU145 = 0.017 ± 0.0062 µM and MDA MB-231 = 0.021 ± 0.0028 µM) than standard against cancer cells presented in Table 1. When one methoxy group is removed, resulting compound **7c** displayed slightly decreased activity on four cell lines (MCF-7 = 0.88 ± 0.073 µM; A549 = 1.44 ± 0.32 µM; DU145 = 1.28 ± 0.27 µM and MDA MB-231 = 1.95 ± 0.19 µM) when compared with **7b**. Where, compound **7d** with 4-methoxy substituent has showed reduced anticancer activity (MCF-7 = 1.78 ± 0.22 µM; A549 = 1.67 ± 0.49 µM; DU145 = 2.10 ± 1.09 µM and MDA MB-231 = 2.34 ± 1.10 µM) than **7c.** Instead of 4-methoxy group with weak electron-donating 4-methyl group (**7i**) exhibited acceptable activity (MCF-7 = 2.17 ± 1.66 µM; A549 = 1.88 ± 0.25 µM; DU145 = 2.65 ± 1.26 µM and MDA MB-231 = 2.14 ± 0.94 µM). Further, the compounds **7e**, **7f**, **7g**, **7h** and **7j**, which possess electron-withdrawing substituents on the phenyl ring, showed moderate activities compared to those compounds **7a**, **7b**, **7c**, **7d** and **7i**, without electron withdrawing and with electron-donating substituents.

### Molecular docking

The docking studies of the potent 1,2,4-Oxadiazole linked 5-Fluorouracil derivatives (**7a**–**7j**) were performed using Molegro Virtual Docker (MVD). The crystal structure of Human VGEFR-2 enzyme (PDB ID: 1YWN) along with the crystal ligand imatinib was downloaded from protein databank [40]. Human VGEFR-2 enzyme is the key enzyme in angiogenesis, hematopoiesis and vasculogenic. All the chemical structures were prepared by using Marvin sketch and minimized and saved in a single file as SDF format. MVD was used to perform computational studies, cavity prediction, assigning bond orders, defining the active binding sites of the Human VGEFR-2 enzyme, structure refinement and preparation. The protein preparation was carried out with MVD and the chain was treated to add missing hydrogen, assign proper bond orders and deleted water molecules. The structure output format was set to pose viewer file so as to view the output of resulting docking studies and hydrogen bond interactions of different poses with the protein. The 2D and 3D interactions were generated by using Discovery Studio Visualizer.

### In silico ADMET prediction

In silico ADMET screened for 5-fluoro-1-(4-(5-substituted phenyl-1,2,4-oxadiazol-3-yl)benzyl)pyrimidine-2,4(1H,3H)-dione derivatives (**7a**–**7j**) was assessed by using DataWarrior Software [41]. It calculates the properties of the datasets of ligands to determine the violation of Lipinski’s rule of 5 and toxicity parameters. All the calculated chemical properties are represented in Table 2.

**Table 2:** In silico ADMET prediction of 1,2,4-oxadiazole linked 5-fluorouracil derivatives (**7a**–**7j**)

Compd	Total Molweight	cLogP	cLogS	HAA	HDA	TSA	Nrotb	TPSA	Druglikeness	Mutagenic	Tumorigenic	Reproductive effective	Irritant
**7a**	364.3	2.40	− 6.49	7	1	268.57	4	88.33	− 1.025	None	None	None	None
**7b**	454.4	2.19	− 6.54	10	1	335.35	7	116.02	− 0.96	None	None	None	None
**7c**	424.3	2.26	− 6.56	9	1	313.09	6	106.79	− 0.96	None	None	None	None
**7d**	394.3	2.33	− 6.50	8	1	290.83	5	97.56	− 0.96	None	None	None	None
**7e**	398.7	3.00	− 7.22	7	1	283.99	4	88.33	− 0.97	None	None	None	None
**7f**	443.2	3.12	− 7.32	7	1	287.2	4	88.33	− 2.81	None	None	None	None
**7g**	409.3	1.48	− 6.95	10	1	292.24	5	134.15	− 6.03	None	None	None	None
**7h**	454.3	0.56	− 7.41	13	1	315.91	6	179.97	− 6.03	None	None	None	None
**7i**	378.3	2.74	− 6.83	7	1	280.83	4	88.33	− 1.05	None	None	None	None
**7j**	533.2	1.28	− 8.24	13	1	334.54	6	179.97	− 7.82	None	None	None	None

### Molecular docking studies

The docking studies of the potent compounds **7a**–**7j** were performed using Molegro Virtual Docker (MVD). The crystal structure of human Vascular Endothelial Growth Factor Receptors (VEGFR-2) enzyme (PDB ID: 1YWN) along with the co-crystal ligand was downloaded from protein databank. Design of VEGFR-2 could be potential target for inhibition of blood vessel formation and leads to the development of anti-angiogenesis agents. All the chemical structures were prepared by using Marvin sketch and minimized. Mol file format structures were converted into Mol2 file format by using Discovery studio. MVD was used to perform computational studies, cavity prediction, assigning bond orders, structure refinement, defining the active binding sites of the VEGFR-2 and structure preparation. The protein preparation was carried out with MVD and the chain was treated to add missing hydrogen, assign proper bond orders and deleted water molecules. The structure output format was set to pose viewer file so as to view the output of resulting docking studies and hydrogen bond interactions of different poses with the protein. The 2D interactions were generated from Protein Plus and 3D interactions were generated from MVD.

**Fig. 2:**
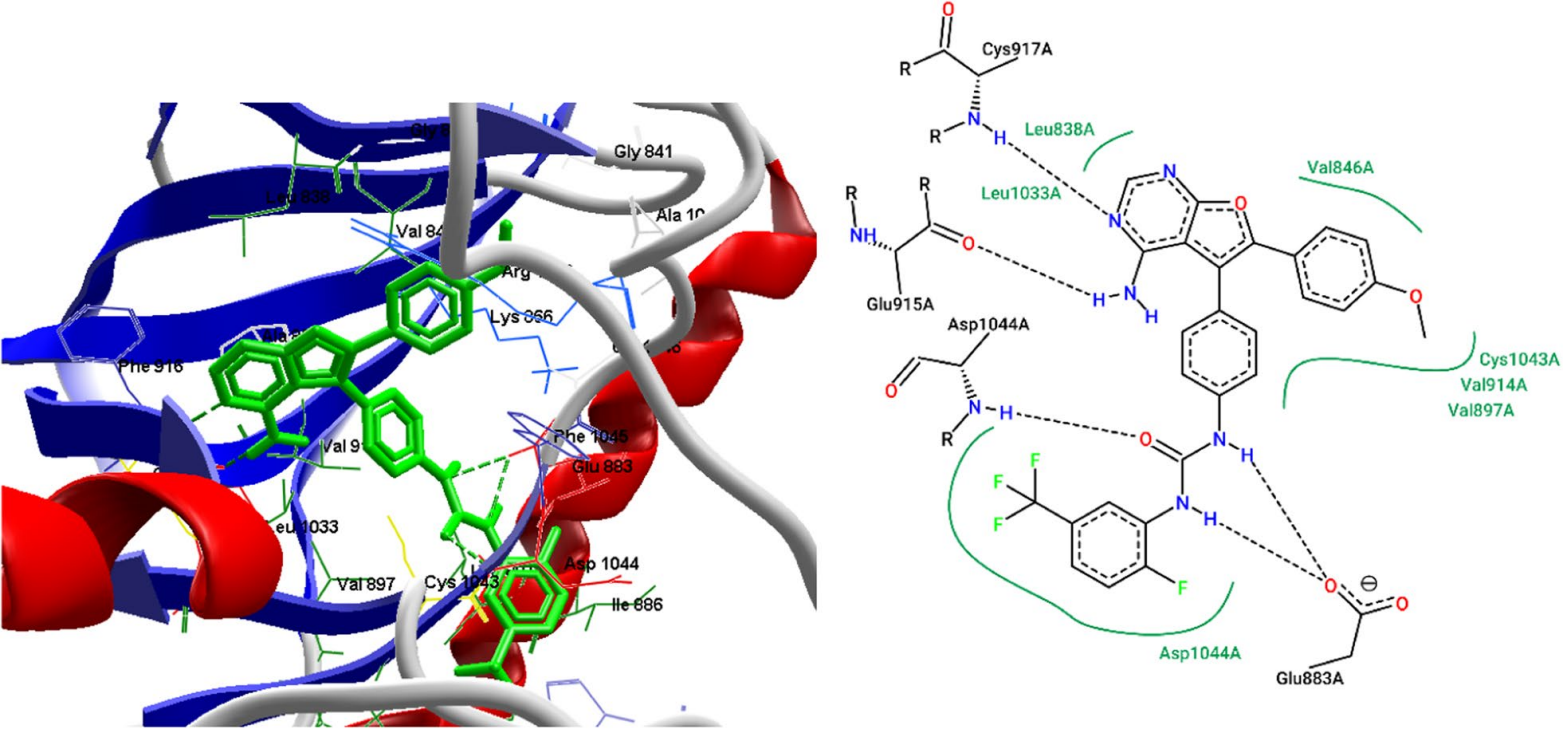
3D and 2D interactions of co-crystal ligand with VEGFR protein (PDB ID: 1YWN)

In the present study, the synthesized compounds were docked into X-ray crystal structures of Human VEGFR-2 enzyme (PDB ID: 1YWN) to understand the possible target mechanism of action. The cavities were detected with MVD and the following are the active residues involved with co-crystal ligand, as it forms hydrogen bond interactions with Glu883, Glu915, Cys917, Asp1044. Arg1049 residues and hydrophobic interactions with Leu838, Val846, Val897, Val914, Cys1043, and Asp1044, residues. The docking has validated and found same interactions with Moldock score − 199.37 and H-bond energy − 6.24 kcal/mol (Fig. 2).

**Fig. 3:**
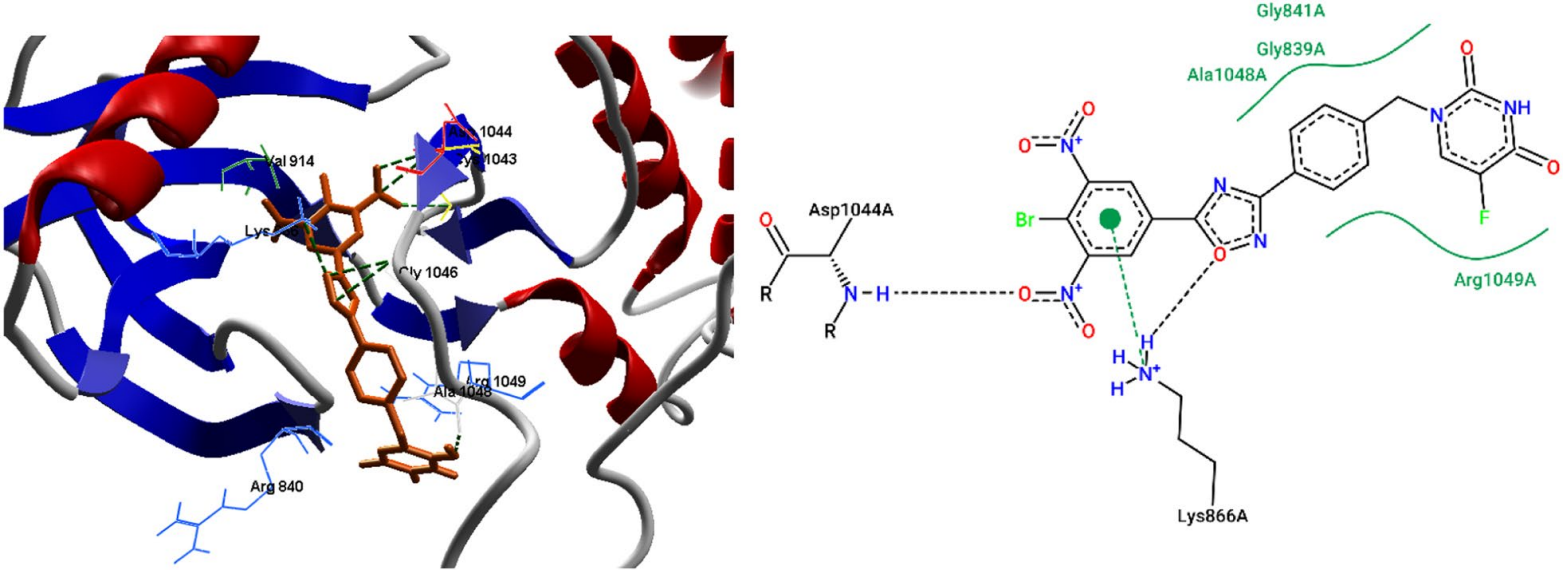
3D and 2D interactions of **7j** with VEGFR protein (PDB ID: 1YWN)

**Fig. 4:**
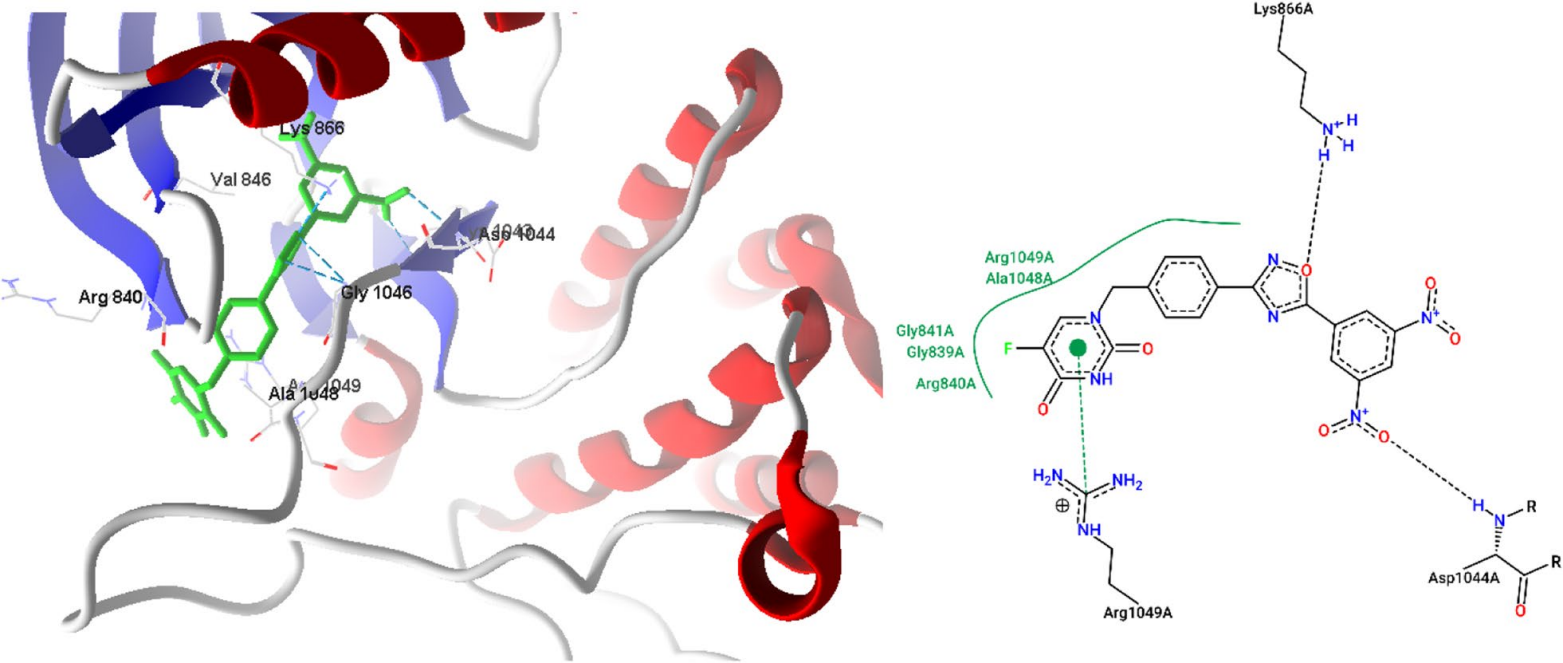
3D and 2D interactions of **7h** with VEGFR protein (PDB ID: 1YWN)

**Fig. 5:**
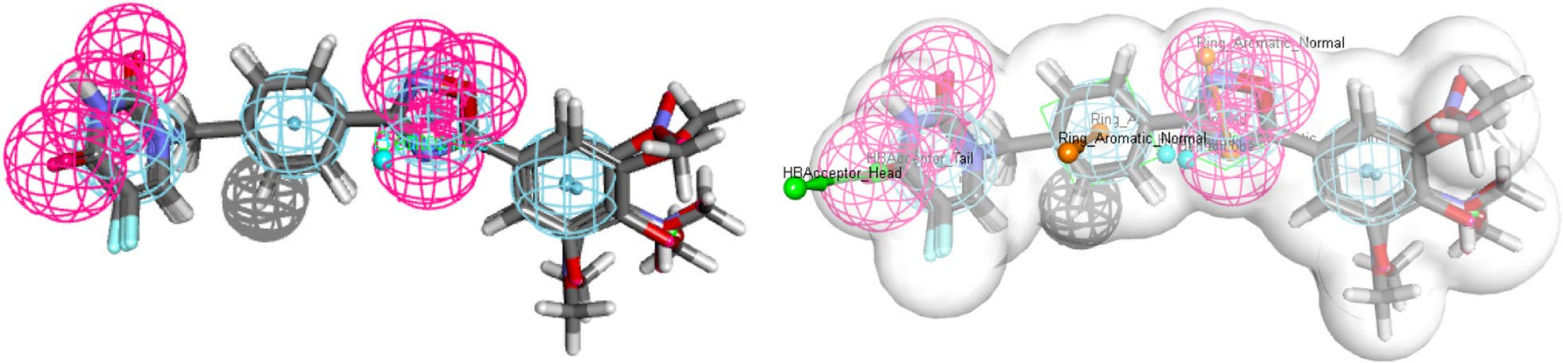
Pharmacophore feature generation by using PharmaGist webserver of all ligands

All the synthesized compounds form hydrogen bond interactions with Asp1044 and have shown same interaction with crystal ligand. The binding affinity of the docked compounds were expressed in negative binding energy kcal/mol (Moldock score). The ligands with more negative value of Moldock score will have more affinity with protein binding. 3,5-dinitro substituted compounds (**7j** and **7h**) established a hydrogen bond (NH—ON) with amino group of Asp1044 and oxygen atom of oxadiazole (NH—O) with amino group of Lys866 with a H-bond energy − 7.40 and − 7.34, respectively and with Moldock score values are − 156.20 and − 157.88, respectively. The **7j** and **7h** also form same hydrophobic interactions with Arg840, Val846, Asp1044, Gly1046, and Arg1049 amino acid residues (Figs. 3, 4). Compound **7c** oxygen of oxadiazole group forms a hydrogen bond (NH—O) with amino hydrogen of Gly1046 with a H-bond energy of − 2.59 kcal/mol (Fig. 5) and hydrophobic interactions with Val846, Arg840, Gly1046, Arg1049 amino acid residues. All the docked ligands have exhibited same interactions with active site of VEGFR. From the data it is revealed that compounds of the series **7j** (Moldock score − 156.20 kcal/mol) and **7h** (Moldock score − 157.88 kcal/mol) showed good inhibitory constant and excellent free energy of binding, which might be the reason for anticancer activity.

### Pharmacophore prediction

All the synthesized compounds generated pharmacophore features by using PharmaGist web server [42]. It will predict the spatial arrangement of features like hydrogen bond acceptor (HBA), hydrogen bond donor (HBD), hydrophobic center (HY) and positive ionisable (PI) which are essential for a ligand to interact with specific target protein. All compounds were submitted to PharmaGist and the pharmacophoric score is found to be 75.17 with 10 Spatial Features, 4 Aromatic, 1 Donor and 5 Acceptor features for all set of ligands.

### In silico ADMET properties

**Fig. 6:**
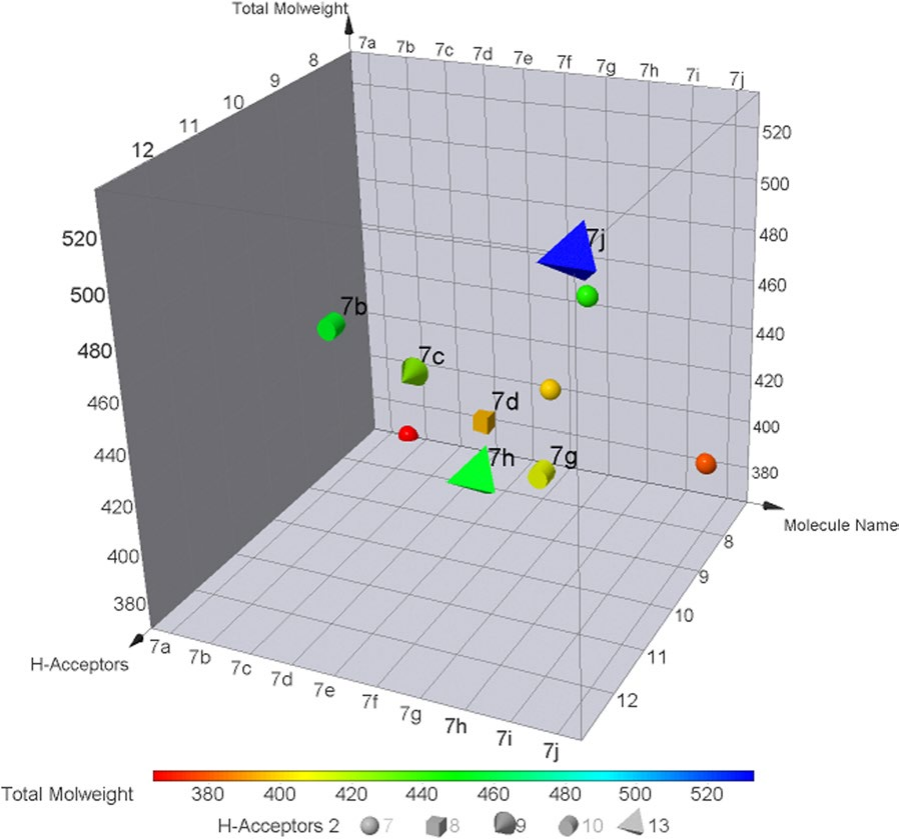
3DPlot of in silico ADMET prediction

The in silico ADMET properties of 5-fluoro-1-(4-(5-substituted phenyl-1,2,4-oxadiazol-3-yl)benzyl)pyrimidine-2,4(1H,3H)-dione derivatives (**7a**–**7j**) was assessed by using DataWarrior Software (Fig. 6). All the compounds were determined molecular descriptors for Rule of 5 (Lipinski rule) which states the oral bioavailability and drug like properties. The determinants possess Molecular weight ≤ 500, no of H-Acceptors ≤ 10, no of H-Donor ≤ 5. Among the calculated chemical descriptors, all the ligands have passed the Lipinski rule which states ligands did not violate more than one Rule of 5, except compound **7j** substituted with 4-bromo-3,5-dinitro has violated Molecular weight and H-Acceptor.

### MTT assay

Individual wells of a 96-well tissue culture micro titer plate were inoculated with 100 µl of complete medium containing 1 × 10^4^ cells. The plates were incubated at 37 °C in a humidified 5% CO_2_ incubator for 18 h prior to the experiment. The results are shown in Table 3.

**Table 3:** Docking score and binding interactions of compounds with VEGFR kinase enzyme into the active site of 1YWN

S. no	Compound	Mol.dock score	Rerank score	H-bond (kcal/mol)	Interactions	
					H-bonding	Steric
1	7a	− 147.97	− 121.95	− 5.01	Lys866, Arg1030	Lys866, Val912, Gly920
2	7b	− 123.29	− 102.36	− 5.01	Lys866, Cys1043, Gly1046	Lys866, Cys1043, Gly1046, Ala1048, Arg1049
3	7c	− 151.14	− 111.31	− 2.59	Lys866, Gly1046	Val846, Arg840, Gly1046, Arg1049
4	7d	− 135.85	− 113.49	− 5.20	Lys866, Gly1046	Arg840, Lys866, Gly1046, Arg1049
5	7e	− 140.55	− 107.94	− 1.03	Arg1030	Val912, Val914, Gly920, Phe1045, Arg1049
6	7f	− 126.70	− 99.40	− 2.44	Arg1030	Val912, Val914, Ile913, Gly920, Arg1030, Phe1045
7	7g	− 124.66	− 98.82	− 2.30	Arg921, Cys1043	Asn921, Cys1043, Phe1045
8	7h	− 157.88	− 116.87	− 7.34	Lys866, Asp1044, Gly1046	Val846, Arg840, Lys866, Cys1043, Asp1044, Ala1048, Arg1049, Gly1046
9	7i	− 141.45	− 113.61	− 2.84	Arg1030	Val912, Asn921, Arg1030, Phe1045, Arg1049
10	7j	− 156.20	− 123.62	− 7.40	Lys866, Cys1043, Asp1044, Gly1046	Arg840, Lys866, Val846, Asp1044, Gly1046, Ala1048, Arg1049
11	4-amino-furo[2,3-d]pyrimidine	− 199.37	156.11	− 6.24	Glu883, Glu915, Cys917, Asp1044 Arg1049	Val846, Val897, Val914, Cys1043, Asp1044

## Conclusion

In conclusion, we have synthesized a library of 1,2,4-oxadiazole linked 5-fluorouracil derivatives (**7a**–**j**) and all these compounds were characterized by ^1^HNMR, ^13^CNMR and Mass spectral analysis. Further, these compounds were investigated for their anticancer activity towards a panel of four human cancer cell lines such as (MCF-7, MDA MB-231), lung cancer (A549) and prostate cancer (DU-145) by using MTT method. Among them, compounds **7a**, **7b**, **7c**, **7d** and **7i** were demonstrated more promising anticancer activity than standard. Further docking studies of all the compounds were presented, where the designed compounds showed good anticancer activity and excellent free energy of binding interactions with VEGFR-2 kinase domain.

## Supplementary Information

**Additional file 1:** Spectra of synthesized compounds.

## Data Availability

All data generated or analyzed during this study are included in this published article (and its Additional file 1).

## References

[1] Ghomi JS, Ghasemzadeh MA (2010). An efficient route to the synthesis of pyrimidine-2-ones under ultrasound irradiation. Dig J Nanomater Biostruct.

[2] Wagner E, Al-Kadasi K, Zimecki M, Sawka-Dobrowolska W (2008). Synthesis and pharmacological screening of derivatives of isoxazolo[4,5-d]pyrimidine. Eur J Med Chem.

[3] Ukrainets IV, Tugaibei IA, Bereznykova NL, Karvechenko VN, Turov AV (2008). 4-Hydroxy-2-quinolones 144. Alkyl-, arylalkyl- and arylamides of 2-hydroxy-4-oxo-4H-pyrido[1,2-a]pyrimidine-3-carboxylic acid and their diuretic properties. Chem Heterocycl Compd.

[4] Ballell L, Field RA, Chung GAC, Young RJ (2007). New thio pyrazolo [3, 4-d] pyrimidine derivatives as anti-mycobacterial agents. Bioorg Med Chem Lett.

[5] Fujiwara N, Nakajima T, Ueda Y, Fujita H, Kawakami H (2008). Novel piperidinylpyrimidine derivatives as inhibitors of HIV-1 LTR activation. Bioorg Med Chem.

[6] Amr AE, Nermien MS, Abdulla MM (2007). Synthesis, reactions, and anti-inflammatory activity of heterocyclic systems fused to a thiophene moiety using citrazinic acid as synthon. Monatsh Chem.

[7] Kurono M, Hayashi M, Miura K, Isogawa Y, Sawai K (1988). A series of substituted pyrimidine has been discovered as a new class of potent antioxidant. Chem Abstr.

[8] Cordeu L, Cubedo E, Bandres E, Rebollo A, Saenz X, Chozas H, Domínguez MV, Echeverria M, Mendivil B, Sanmartin C, Palop JA, Font M, García-Foncillas J (2007). Biological profile of new apoptotic agents based on 2,4-pyrido[2,3-d] pyrimidine derivatives. Bioorg Med Chem.

[9] Prakash O, Kumar R, Kumar R, Tyagi P, Kuhad RC (2007). Organo iodine(III) mediated synthesis of 3,9-diaryl- and 3,9-difuryl-bis-1,2,4-triazolo[4,3-*a*][4,3-*c*]pyrimidines as antibacterial agents. Eur J Med Chem.

[10] Katiyar SB, Bansal I, Saxena JK, Chauhan PMS (2005). Syntheses of 2,4,6-trisubstituted pyrimidine derivatives as a new class of anti-filarial topoisomerase II inhibitors. Bioorg Med Chem Lett.

[11] Patel RB, Desai PS, Desai KR, Chikhalia KH (2006). Synthesis of pyrimidine based thiazolidinones and azetidinones. Antimicrobial and antitubercular agents. Indian J Chem.

[12] Cox RA. Quart Rev. 1968;22:934.

[13] Callery P, Gannett P, Williams DA, Lemke TL (2002). Cancer and cancer chemotherapy. Foye’s principles of medicinal chemistry.

[14] Heidelberger C, Chaudhuri NK, Danneberg P, Mooren D, Griesbach L, Duschinsky R, Schnitzer RJ, Pleven E, Sheiner J (1957). Fluorinated pyrimidines, a new class of tumour-inhibitory compounds. Nature.

[15] Dando EE, Lim GFS, Lim SJM (2020). Intralesional 5-fluorouracil for the nonsurgical management of low-risk, invasive squamous cell carcinoma. Dermatol Surg.

[16] Moorkoth D, Nampoothiri KM, Nagarajan S, Girija AA, Balasubramaniyan S, Sakthi KD (2021). Star-shaped polylactide dipyridamole conjugated to 5-fluorouracil and 4-piperidinopiperidine nanocarriers for bioimaging and dual drug delivery in cancer cells. ACS Appl Polym Mater.

[17] Noordhuis P, Holwerda U, Van der Wilt CL, Van Groeningen CJ, Smid K, Meijer S, Pinedo HM, Peters GJ (2004). 5-Fluorouracil incorporation into RNA and DNA in relation to thymidylate synthase inhibition of human colorectal cancers. Ann Oncol.

[18] Danesi CC, Dihl RR, Bellagamba CB, Andrade RHH, Cunha SK, Guimarães NN, Lehmann M (2012). Genotoxicity testing of combined treatment with cisplatin, bleomycin, and 5-fluorouracil in somatic cells of Drosophila melanogaster. Mut Res.

[19] Metterle L, Nelson C, Patel N (2015). Intralesional 5-fluorouracil (FU) as a treatment for nonmelanoma skin cancer (NMSC): a review. J Am Acad Dermatol.

[20] Rossi S (2013). Australian medicines handbook.

[21] Wood PA, Du-Quiton J, You S, Hrushesky WJ (2006). Circadian clock coordinates cancer cell cycle progression, thymidylate synthase, and 5-fluorouracil therapeutic index. Mol Cancer Ther.

[22] Zhang N, Yin Y, Xu SJ, Chen WS (2008). 5-Fluorouracil: mechanisms of resistance and reversal strategies. Molecules.

[23] Malet-Martino M, Martino R (2002). Clinical studies of three oral prodrugs of 5-fluorouracil (capecitabine, UFT, S-1): a review. Oncologist.

[24] Malet-Martino M, Jolimaitre P, Martino R (2002). The prodrugs of 5-fluorouracil. Curr Med Chem.

[25] Carbone M, Li Y, Irace C, Mollo E, Castelluccio F, Pascale AD, Cimino G, Santamaria R, Guo YW, Gavagnin M (2011). Structure and cytotoxicity of phidianidines A and B: first finding of 1,2,4-oxadiazole system in a marine natural product. Org Lett.

[26] Khan I, Ibrar A, Abbas N (2014). Oxadiazoles as privileged motifs for promising anticancer leads: recent advances and future prospects. Arch Pharm (Weinheim).

[27] Zarghi A, Hajimahdi Z (2013). Substituted oxadiazoles: a patent review (2010–2012). Expert OpinTher Pat.

[28] Pace A, Buscemi S, Piccionello AP, Pibiri I (2015). Recent advances in the chemistry of 1,2,4 oxadiazoles. Adv Heterocycl Chem.

[29] Vaidya A, Jain S, Jain P, Jain P, Tiwari N, Jain R, Jain AK, Agrawal RK (2016). Synthesis and biological activities of oxadiazole derivatives: a review. Mini Rev Med Chem.

[30] Lueg C, Schepmann D, Günther R, Brust P, Wünsch B (2013). Development of fluorinated CB2 receptor agonists for PET studies. Bioorg Med Chem..

[31] Bokach NA, Khripoun AV, Kukushkin VY, Haukka M, Pombeiro AJL (2003). A route to 1,2,4-oxadiazoles and their complexes via platinum-mediated 1,3-dipolar cycloaddition of nitrile oxides to organonitriles. Inorg Chem.

[32] Manfredini S, Lampronti I, Vertuani S, Solaroli N, Recanatini M, Bryan D, McKinney M (2000). Design, synthesis and binding at cloned muscarinic receptors of N-[5-(1'-substituted-acetoxymethyl)-3-oxadiazolyl] and N-[4-(1'-substituted-acetoxymethyl)-2-dioxolanyl] dialkyl amines. Bioorg Med Chem.

[33] Boys ML, Schretzman LA, Chandrakumar NS, Tollefson MB, Mohler SB, Downs VL, Penning TD, Russell MA, Wendt JA, Chen BB, Stenmark HG, Wu H, Spangler DP, Clare M, Desai BN, Khanna IK, Nguyen MN, Duffin T, Engleman VW, Finn MB, Freeman SK, Hanneke ML, Keene JL, Klover JA, Nickols GA, Nickols MA, Steininger CN, Westlin M, William W, Yi XY, Wang Y, Dalton CR, Norring SA (2006). Convergent, parallel synthesis of a series of beta-substituted 1,2,4-oxadiazole butanoic acids as potent and selective alpha(v)beta3 receptor antagonists. Bioorg Med Chem Lett.

[34] dos Santos Filho JM, Leite ACL, de Oliveira BG, Moreira DRM, Lima MS, Soares MBP, Leite LFCC (2009). Design, synthesis and cruzain docking of 3-(4-substituted-aryl)-1,2,4- oxadiazole-N-acylhydrazones as anti-Trypanosoma cruzi agents. Bioorg Med Chem.

[35] de Mel SJ, Sobral AD, Lopes HDL, Srivastava RM (1998). Synthesis of Some 3-aryl-1,2,4-oxadiazoles carrying a protected l-alanine side chain. J Braz Chem Soc.

[36] Clitherow JW, Beswick P, Irving WJ, Scopes DIC, Barnes JC, Clapham J, Brown JD, Evans DJ, Hayes AG (1996). Novel 1, 2, 4-oxadiazoles as potent and selective histamine H3 receptor antagonists. Bioorg Med Chem Lett.

[37] Ispikoudi M, Amvrazis M, Kontogiorgis C, Koumbis AE, Litinas KE, Hadjipavlou-Litina D, Fylaktakidou KC (2010). Convenient synthesis and biological profile of 5-amino-substituted 1,2,4-oxadiazole derivatives. Eur J Med Chem.

[38] Chawla G (2018). 1,2,4-oxadiazole as a privileged scaffold for anti-inflammatory and analgesic activities: a review. Mini Rev Med Chem.

[39] Vaidya A, Jain S, Prashantha KB (2020). Synthesis of 1,2,4-oxadiazole derivatives: anticancer and 3D QSAR studies. Monatsh Chem.

[40] Miyazaki Y, Maeda Y, Sato H, Nakano M, Mellor GW (2008). Rational design of 4-amino-5,6-diaryl-furo[2,3-d]pyrimidines as potent glycogen synthase kinase-3 inhibitors. Bioorg Med Chem Lett.

[41] Sander T, Freyss J, von Korff M, Rufener C (2015). DataWarrior, An open-source program for chemistry aware data visualization andanalysis. J Chem Inf Model.

[42] Inbar Y, Schneidman-Duhovny D, Dror O, Nussinov R, Wolfson HJ (2007). Deterministic pharmacophore detection via multiple flexible alignment ofdrug-like molecules. Annual international conference on research in computational molecular biology.

