# Supplementary material for: Synthesis, biological evaluation and docking studies of 1,2,4-oxadiazole linked 5-fluorouracil derivatives as anticancer agents
Source: BMC Chem. 2021 May 4;15(1):30. doi: 10.1186/s13065-021-00757-y (PMC8097950; doi:10.1186/s13065-021-00757-y)
Supplement: Supplementary file 1 — Additional file 1: Spectra of synthesized compounds. [file 13065_2021_757_MOESM1_ESM.doc]

**Synthesis, Biological Evaluation and Docking studies of 1,2,4-Oxadiazole Linked 5-Fluorouracil Derivatives as Anticancer Agents**

Ravi Kumar B ^[a]^, Shashikala K ^[b]^, G. Rajeshwar Reddy ^[c]^ and Laxminarayana E*^[a]^

[a]Sreenidhi Institute of Science and Technology (Autonomous) Yamnampet, Ghatkesar, Hyderabad,Telangana

Email: elxnkits@yahoo.co.in

[b]Geethanjali College of Engineering and Technology, (Autonomous) Cheeryal, Keesara, Hyderabad, Telangana

[c]University of North Carolina Wilmington, Wilmington, North Carolina, 28409, USA


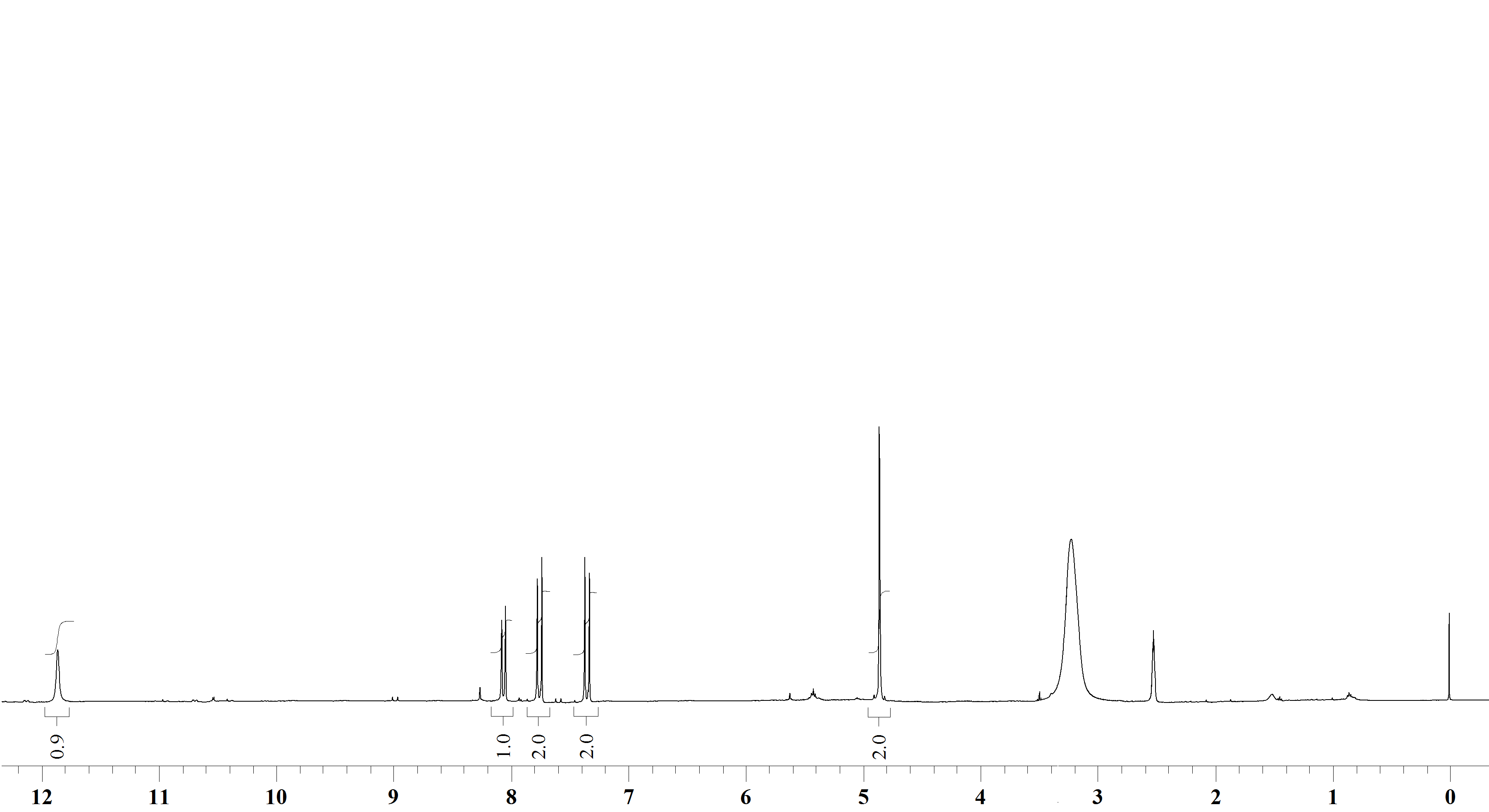


^1^H NMR Spectrum of **4** (300 MHz, DMSO-d6)


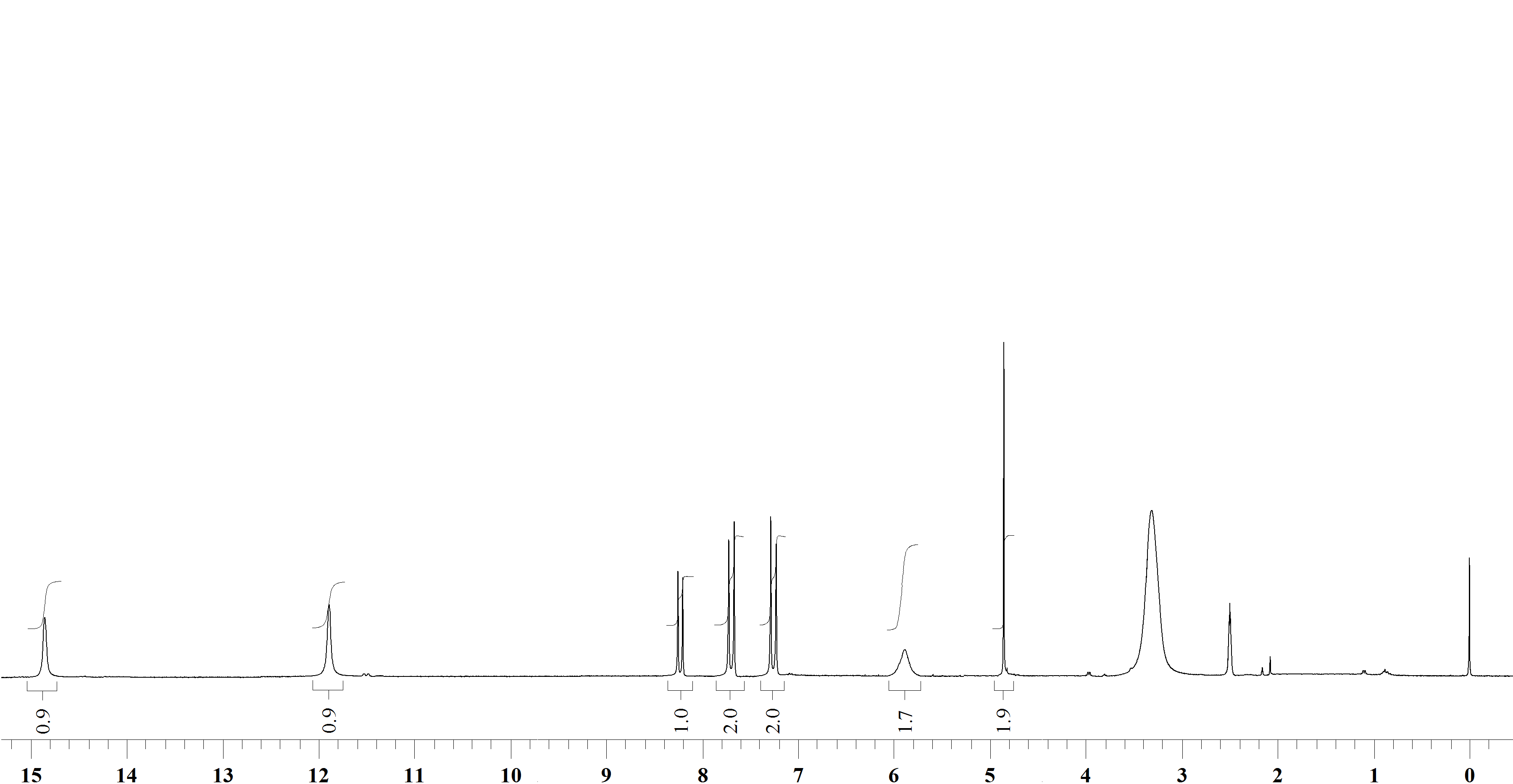


^1^H NMR Spectrum of **5** (300 MHz, DMSO-d6)


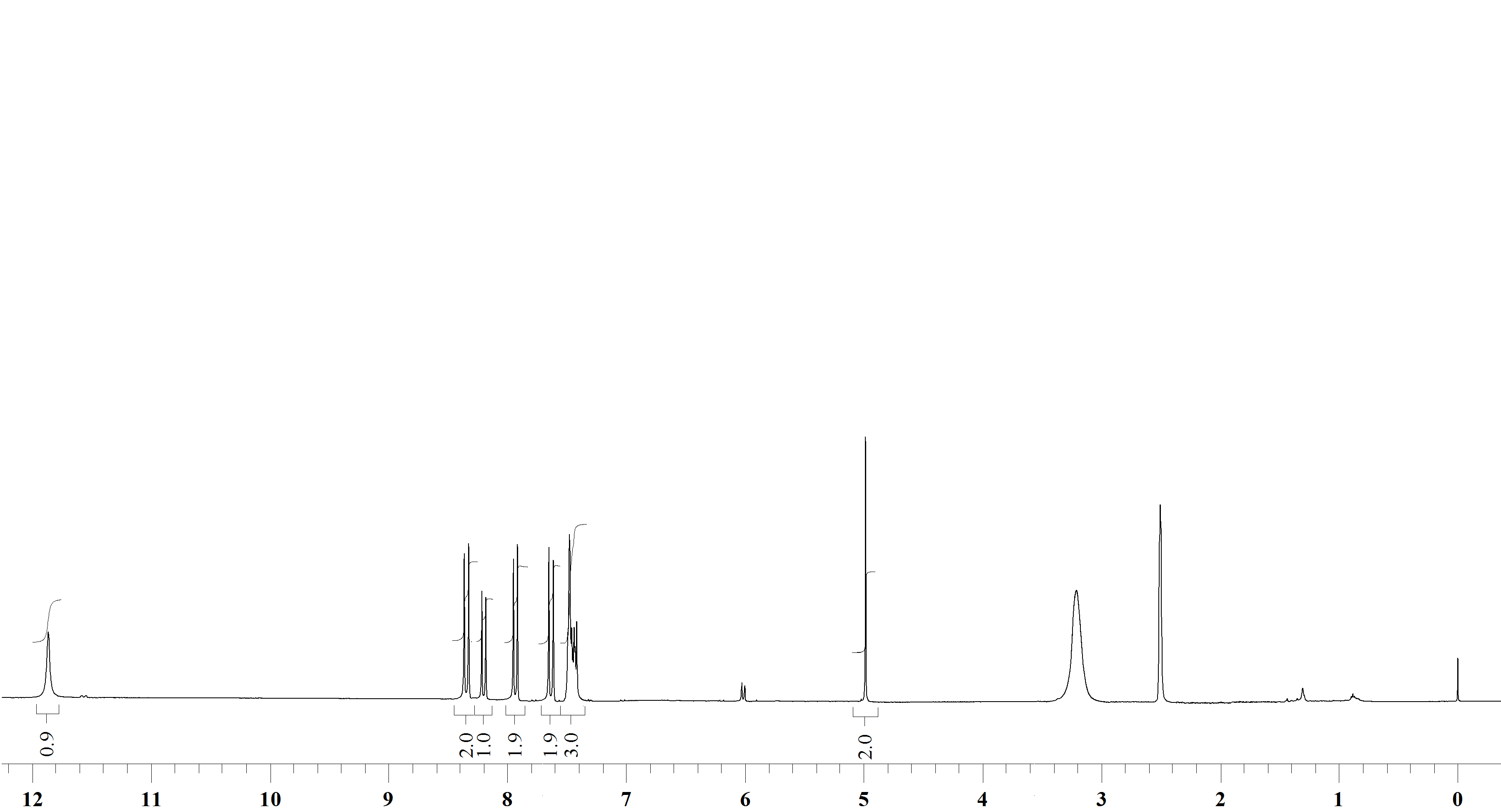


^1^H NMR Spectrum of **7a** (300 MHz, DMSO-d6)


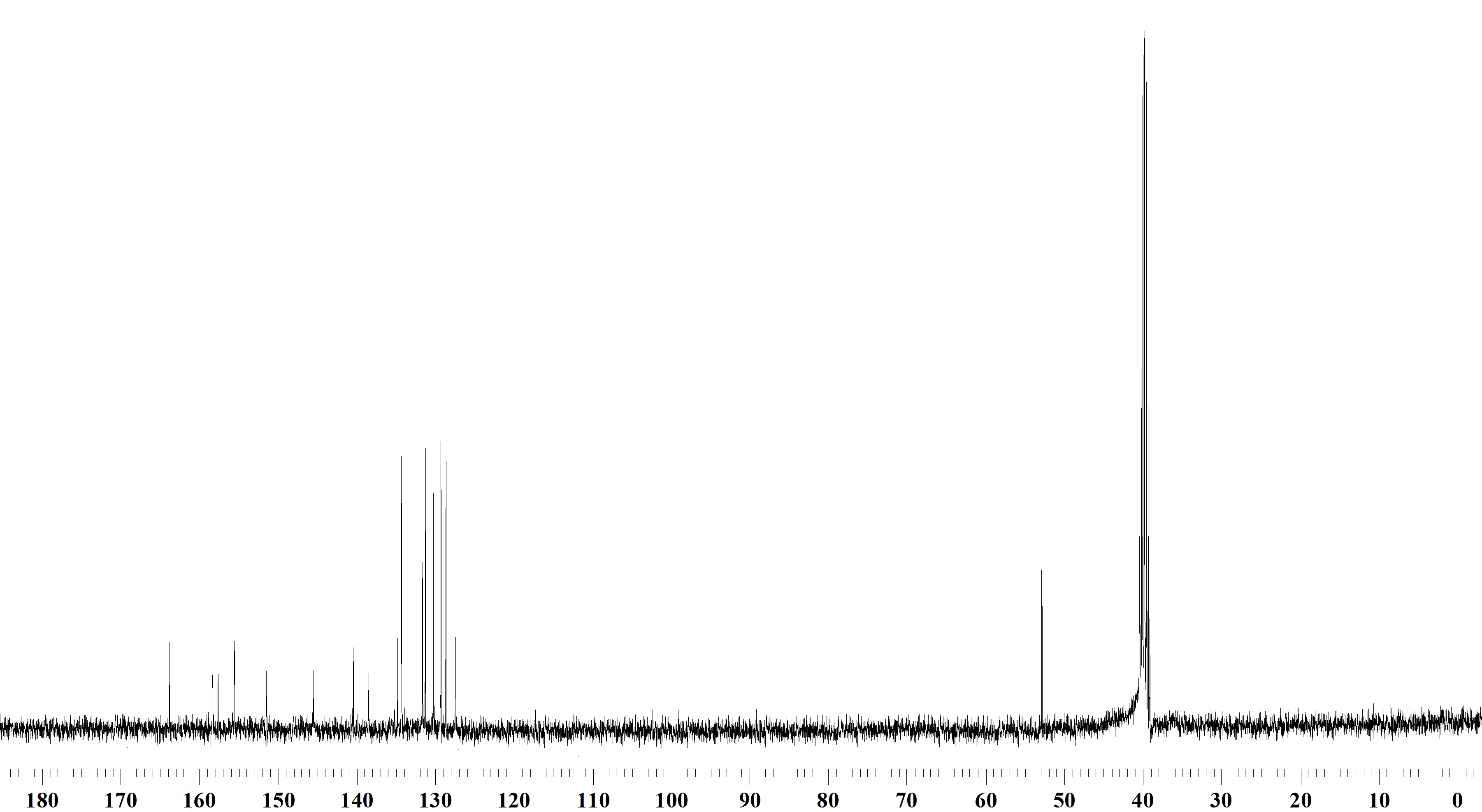


^13^C NMR Spectrum of **7a** (75 MHz, DMSO-d6)


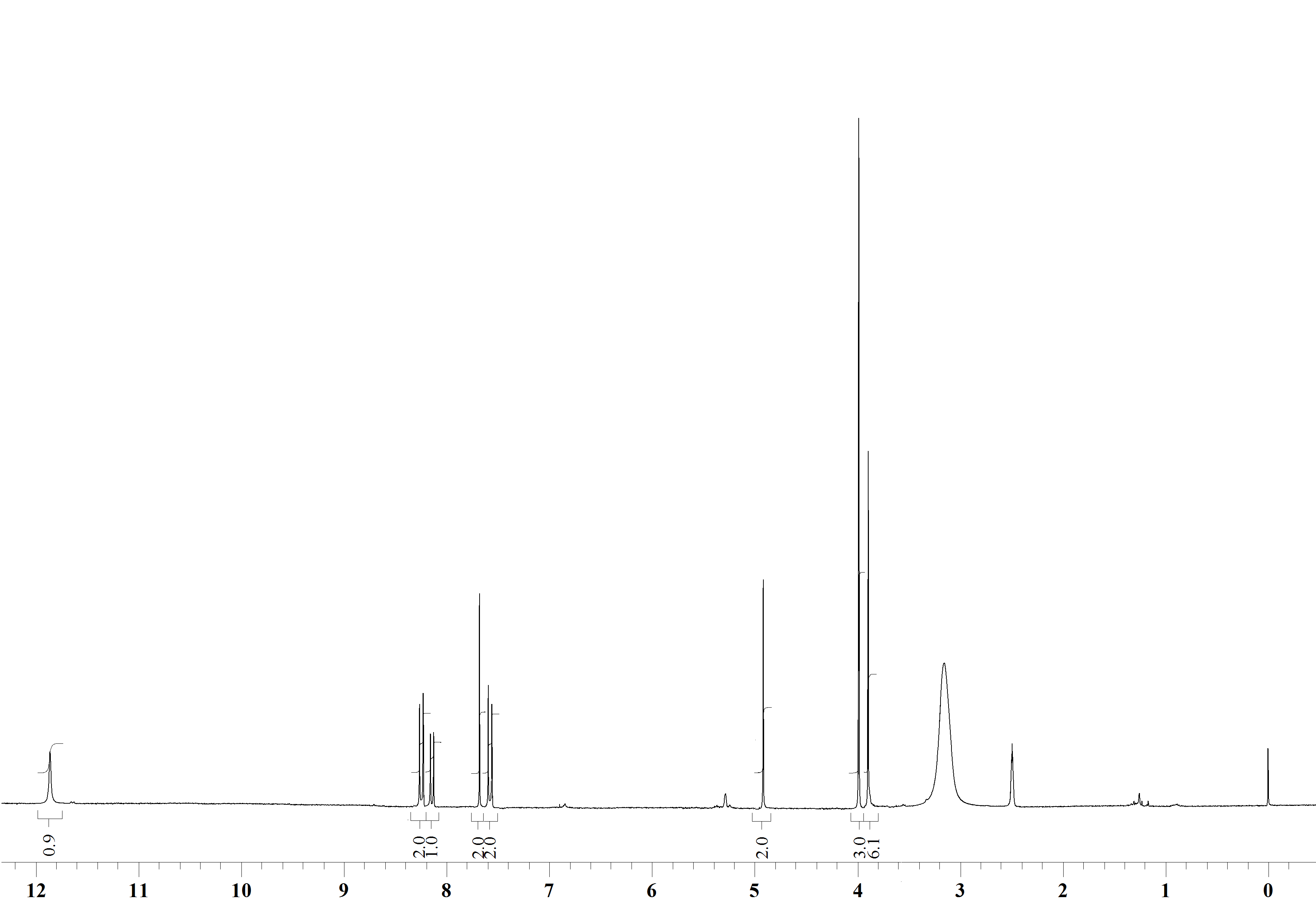


^1^H NMR Spectrum of **7b** (300 MHz, DMSO-d6)


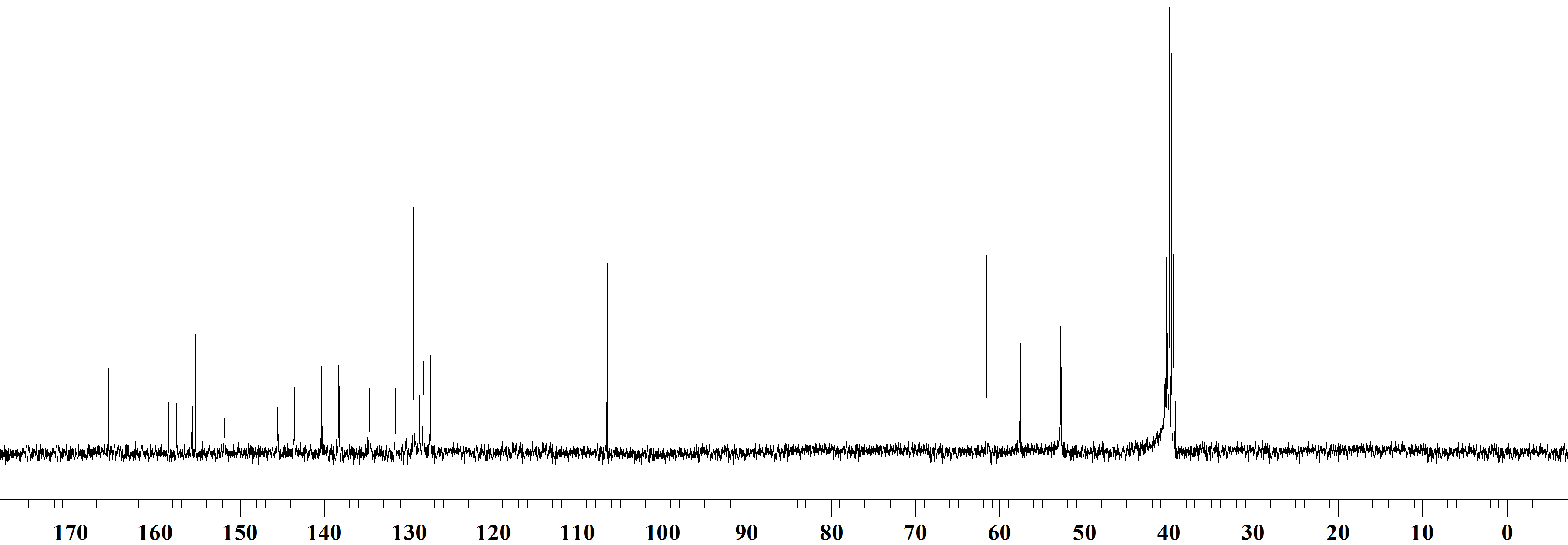


^13^C NMR Spectrum of **7b** (75 MHz, DMSO-d6)


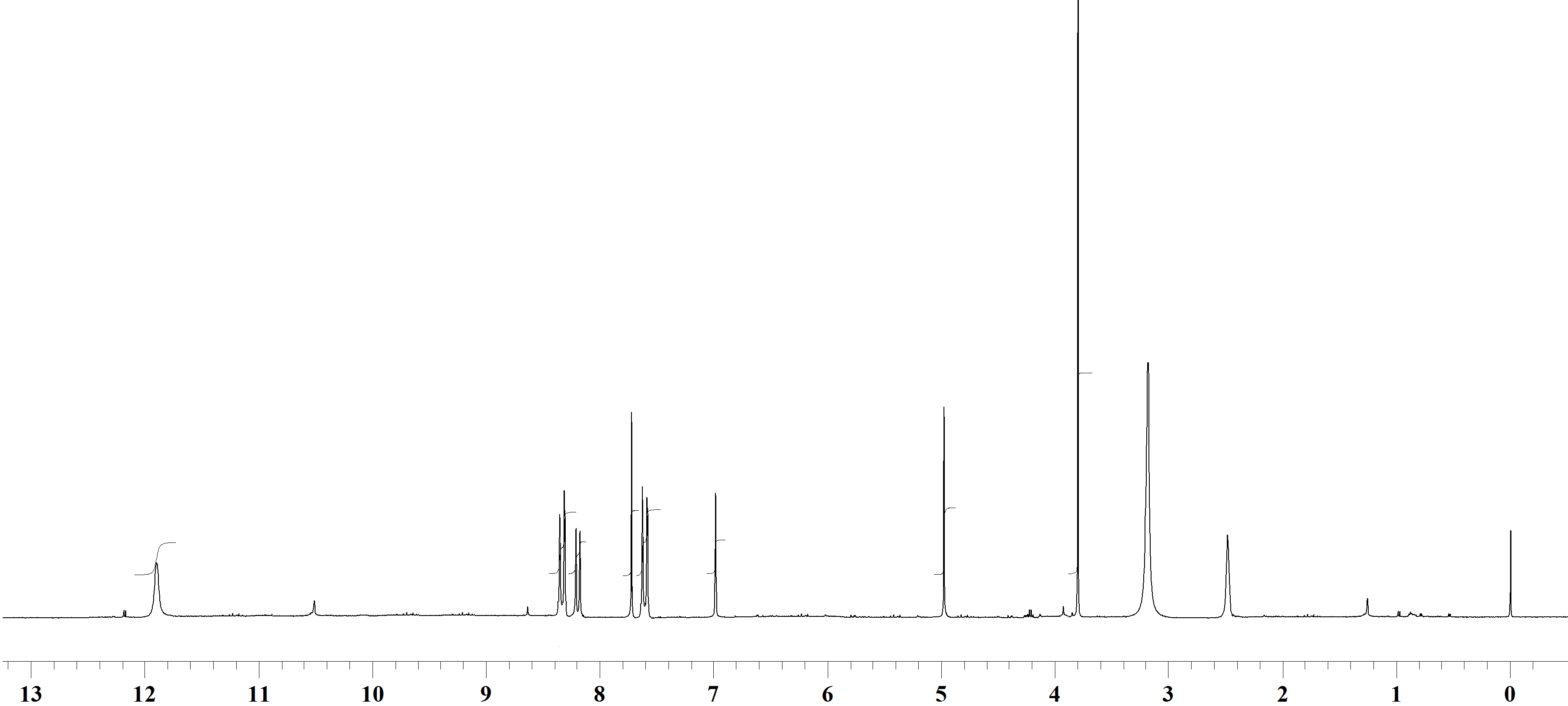


^1^H NMR Spectrum of **7c** (300 MHz, DMSO-d6)


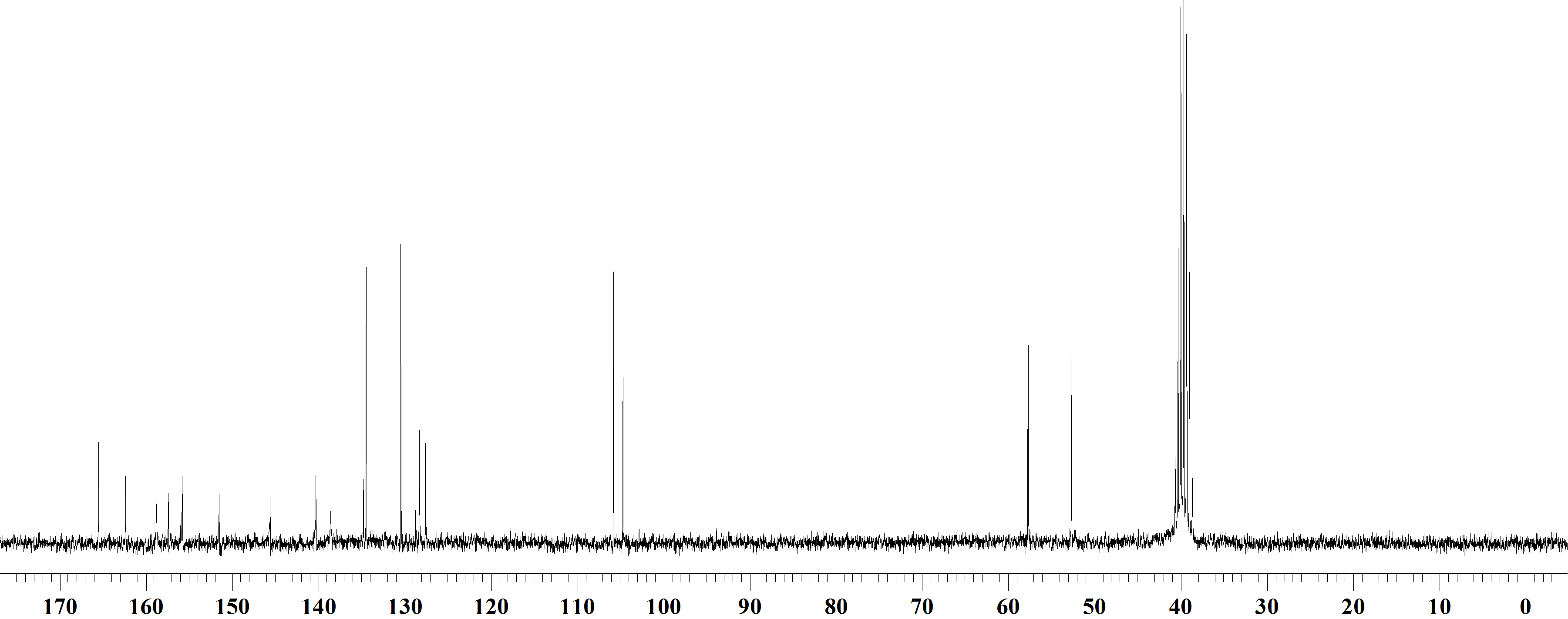


^13^C NMR Spectrum of **7c** (75 MHz, DMSO-d6)


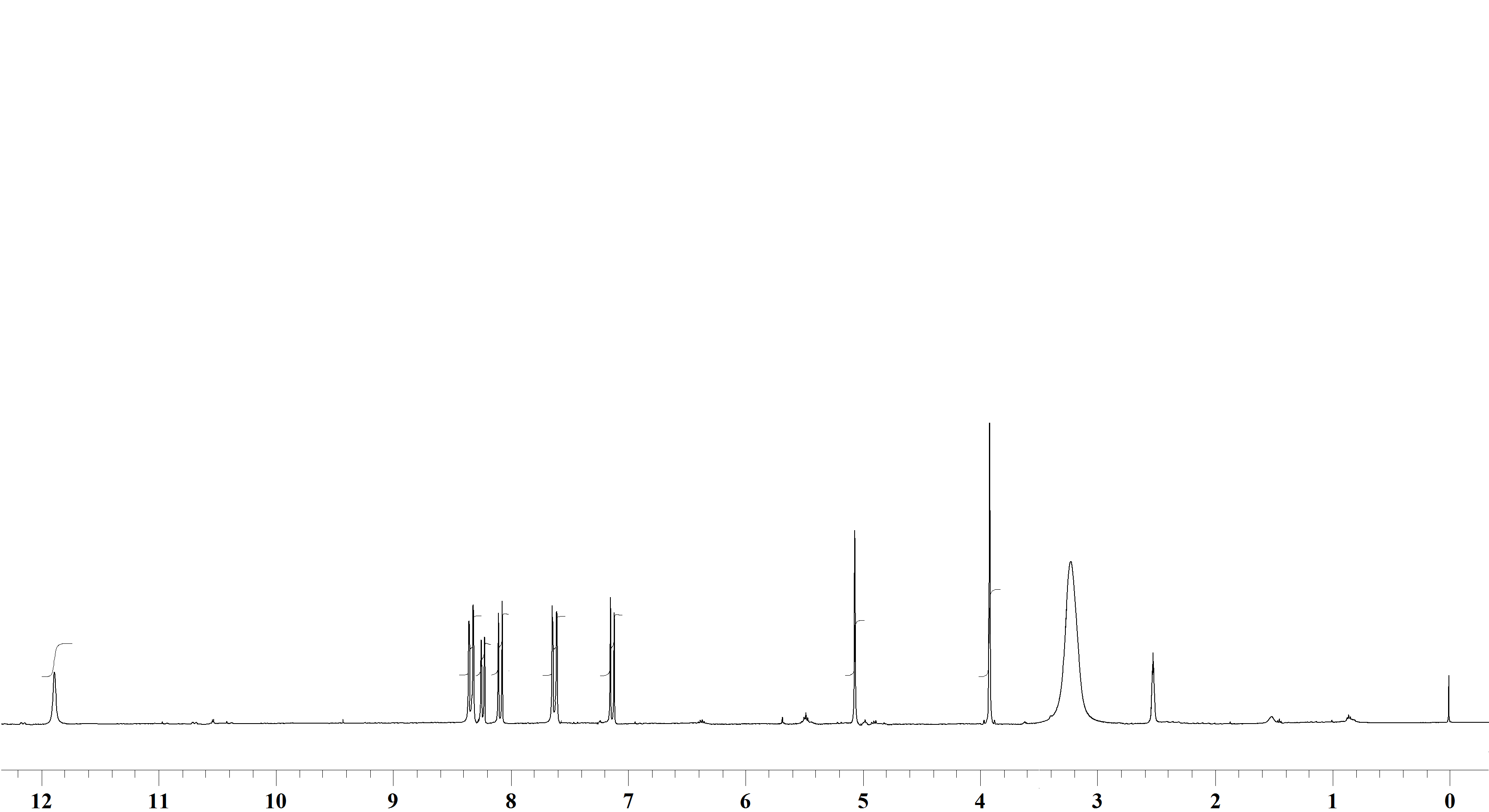


^1^H NMR Spectrum of **7d** (300 MHz, DMSO-d6)


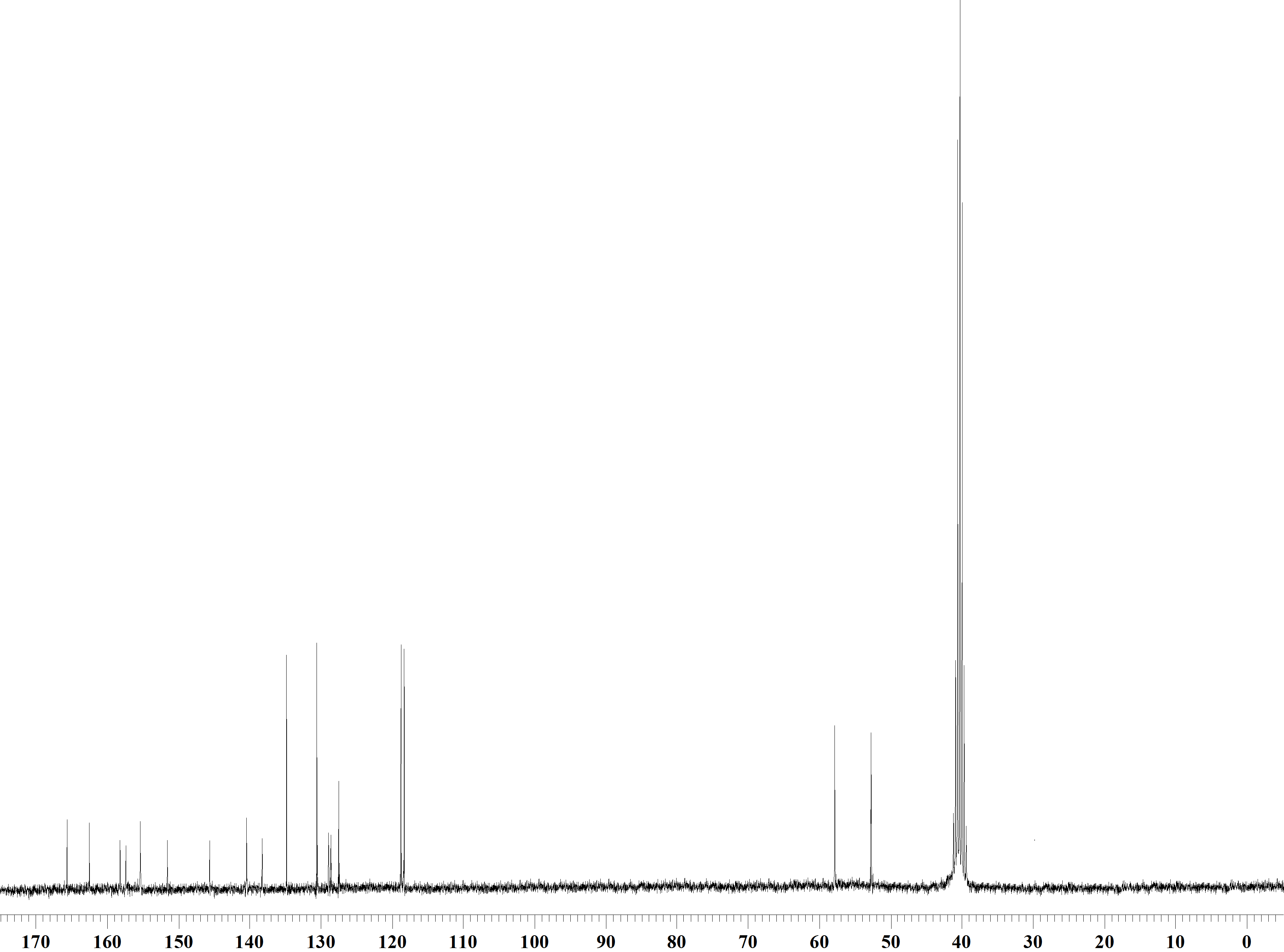


^13^C NMR Spectrum of **7d** (75 MHz, DMSO-d6)


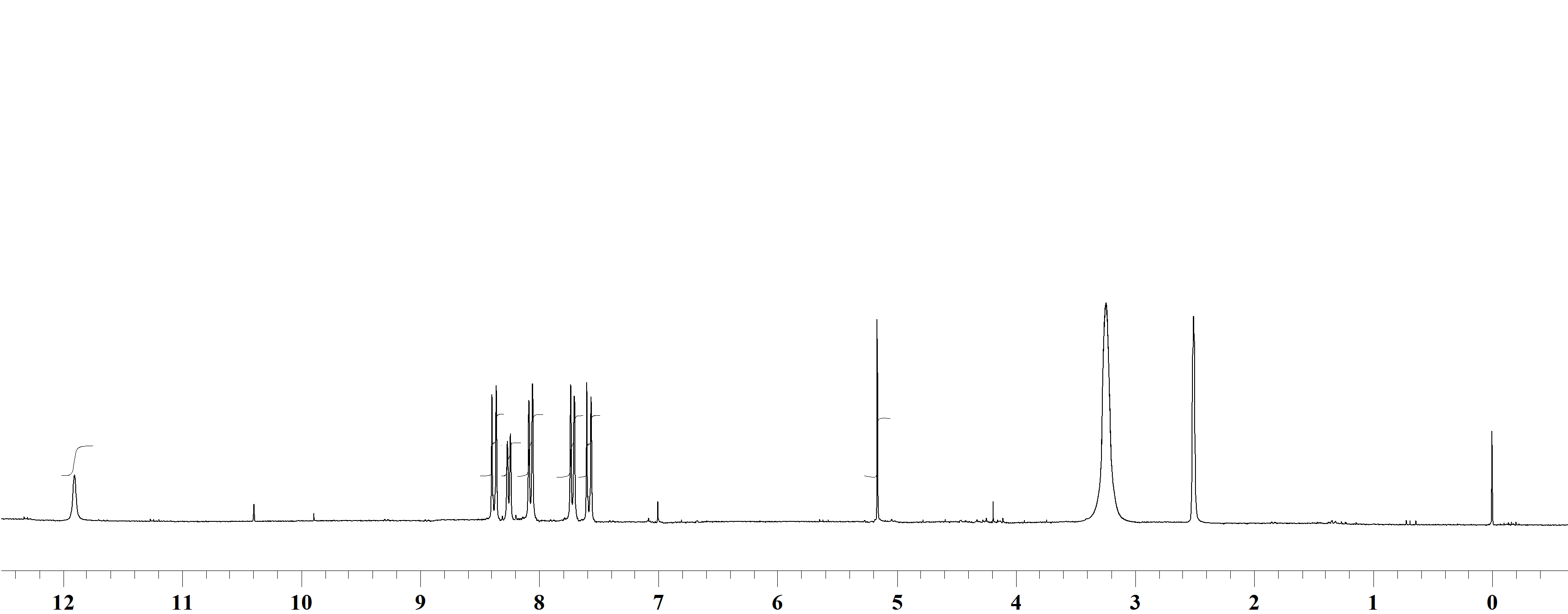


^1^H NMR Spectrum of **7e** (300 MHz, DMSO-d6)


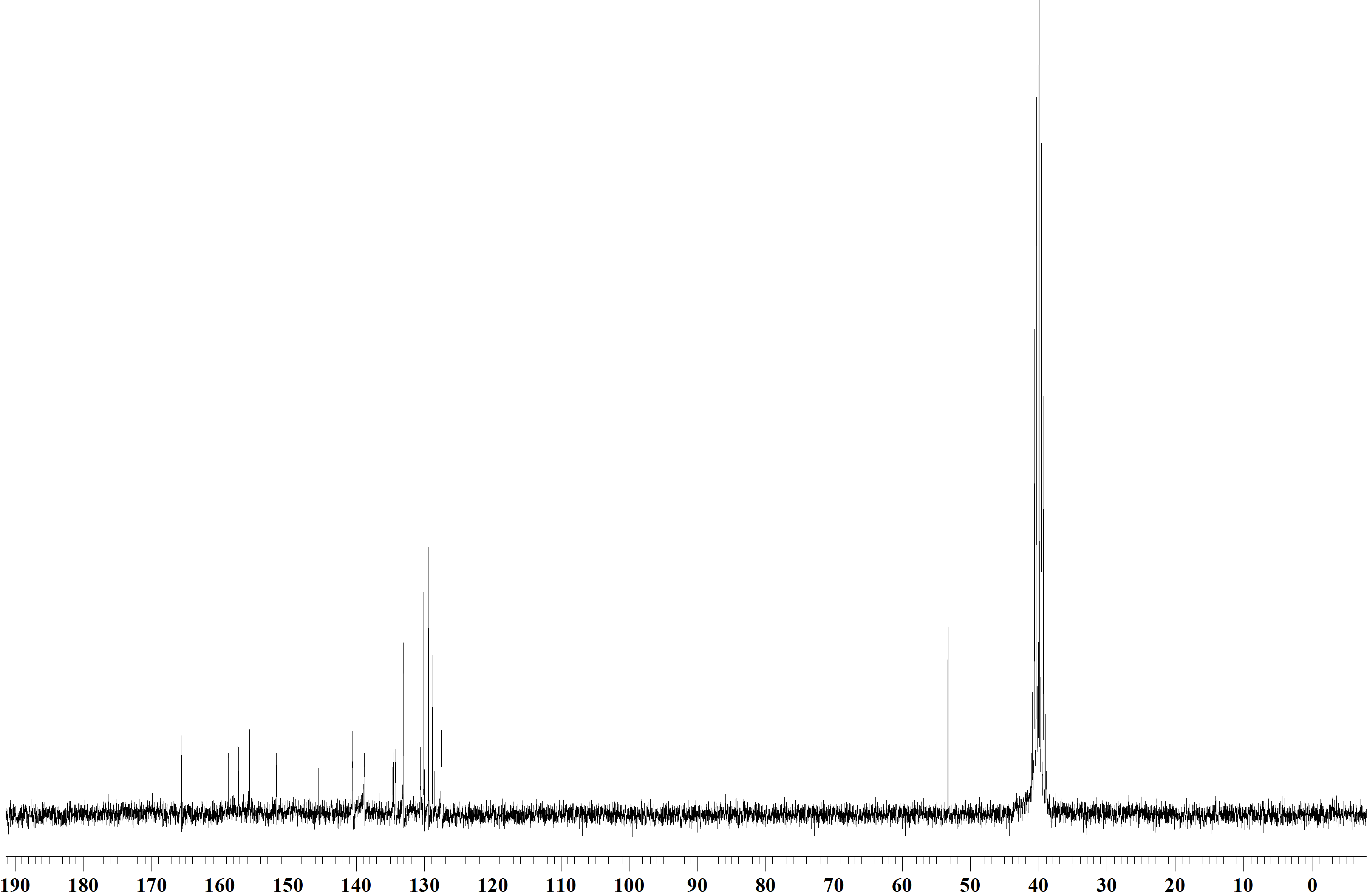


^13^C NMR Spectrum of **7e** (75 MHz, DMSO-d6)


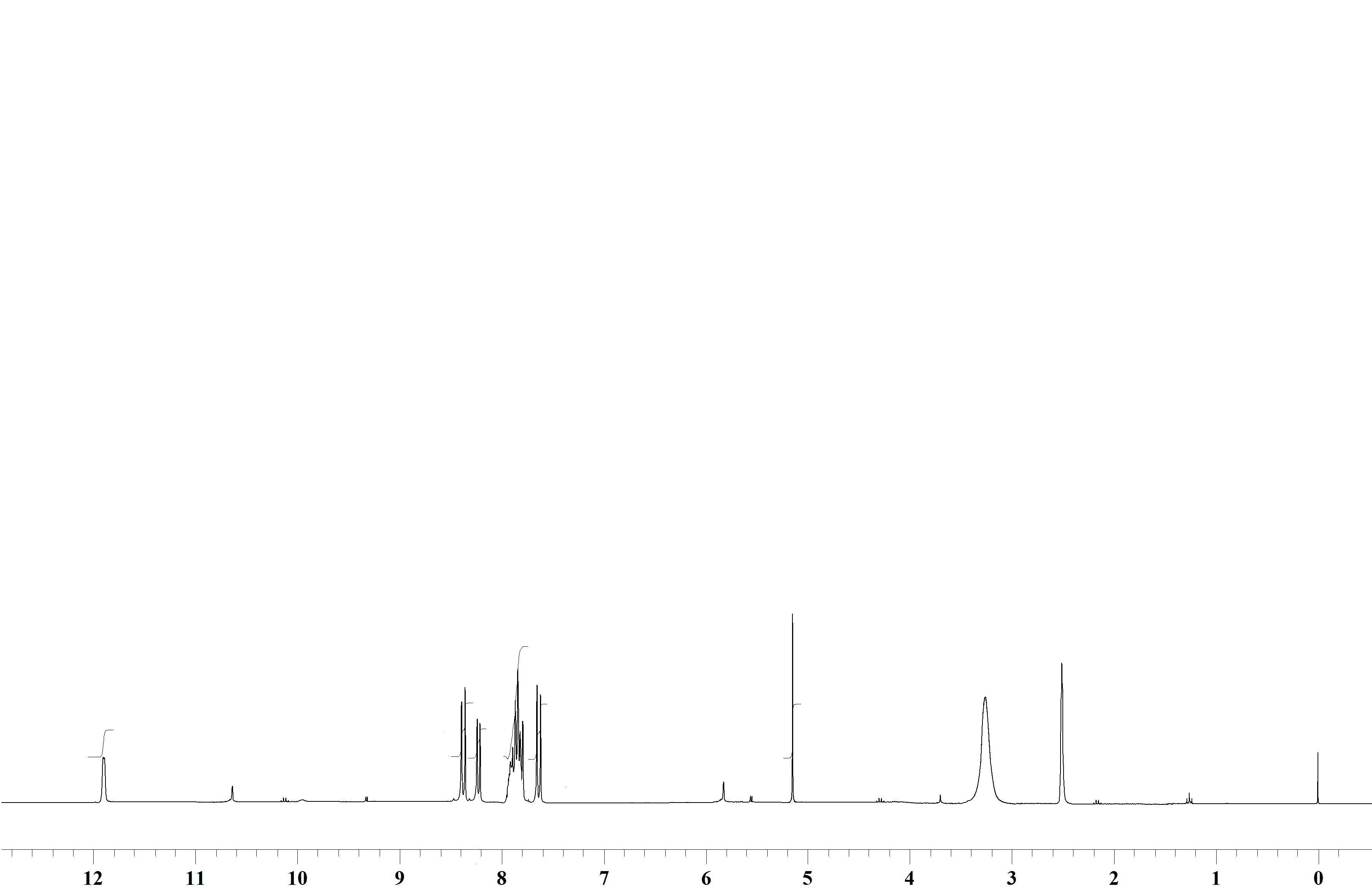


^1^H NMR Spectrum of **7f** (300 MHz, DMSO-d6)


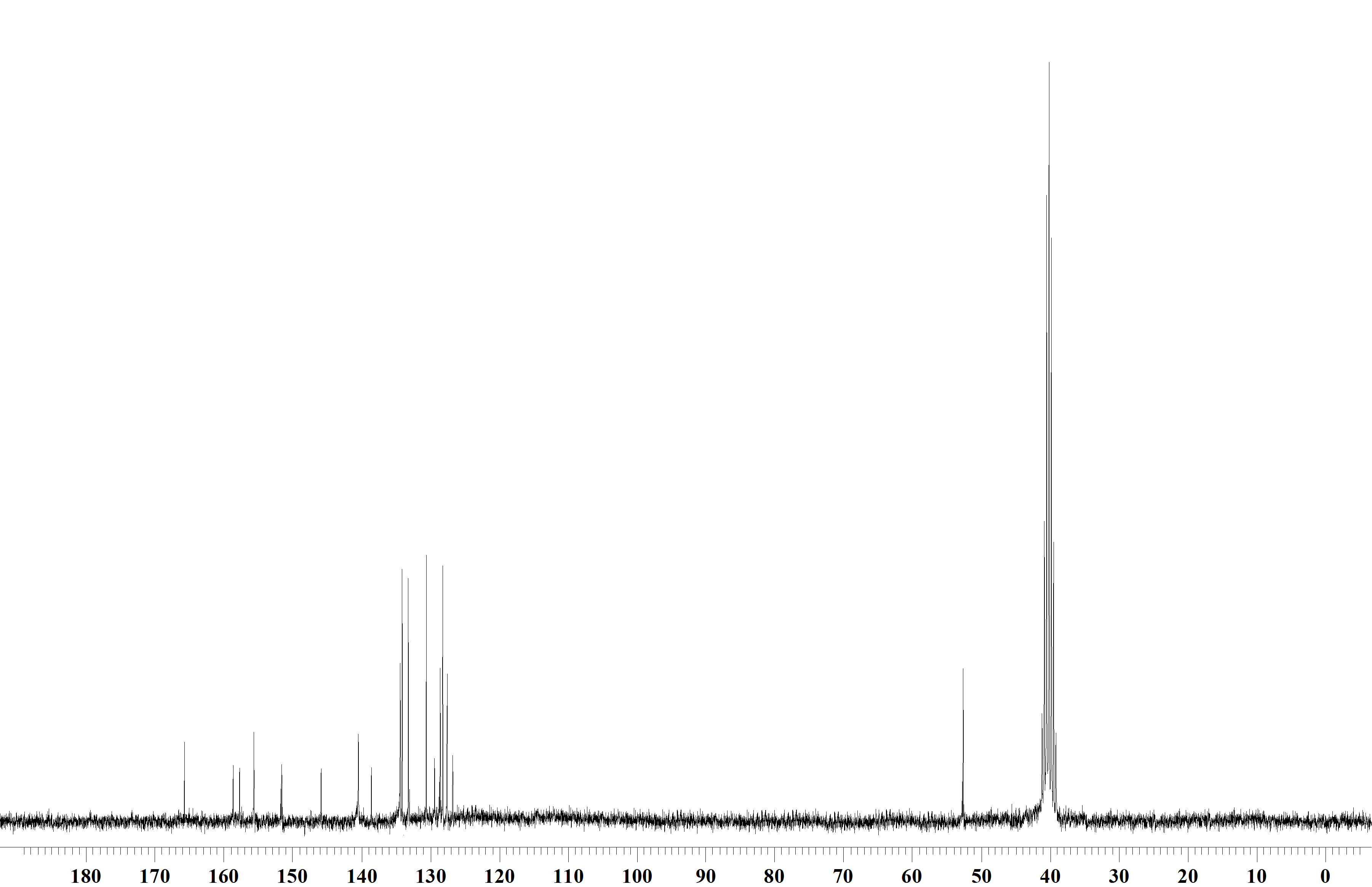


^13^C NMR Spectrum of **7f** (75 MHz, DMSO-d6)


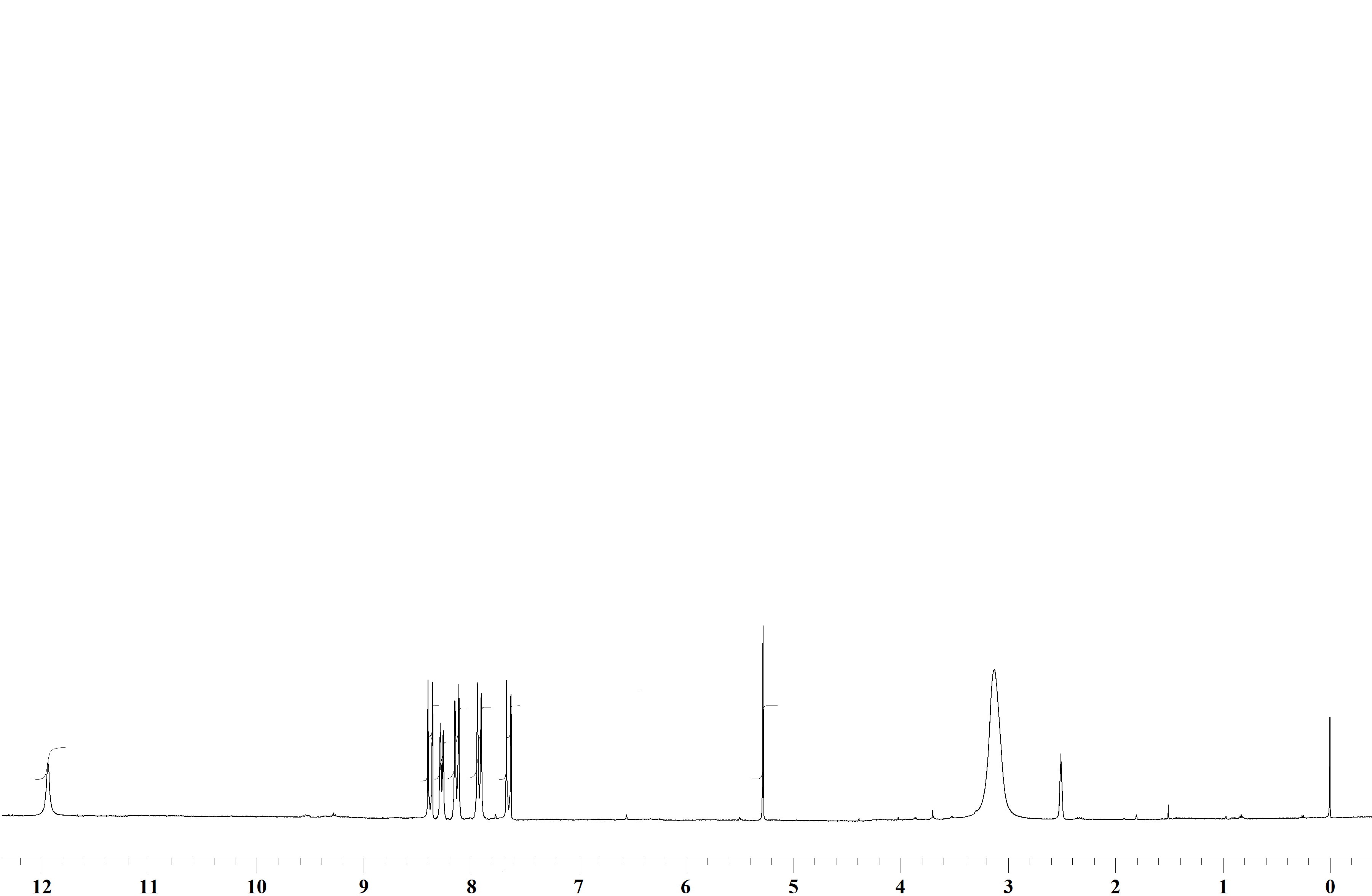


^1^H NMR Spectrum of **7g** (300 MHz, DMSO-d6)


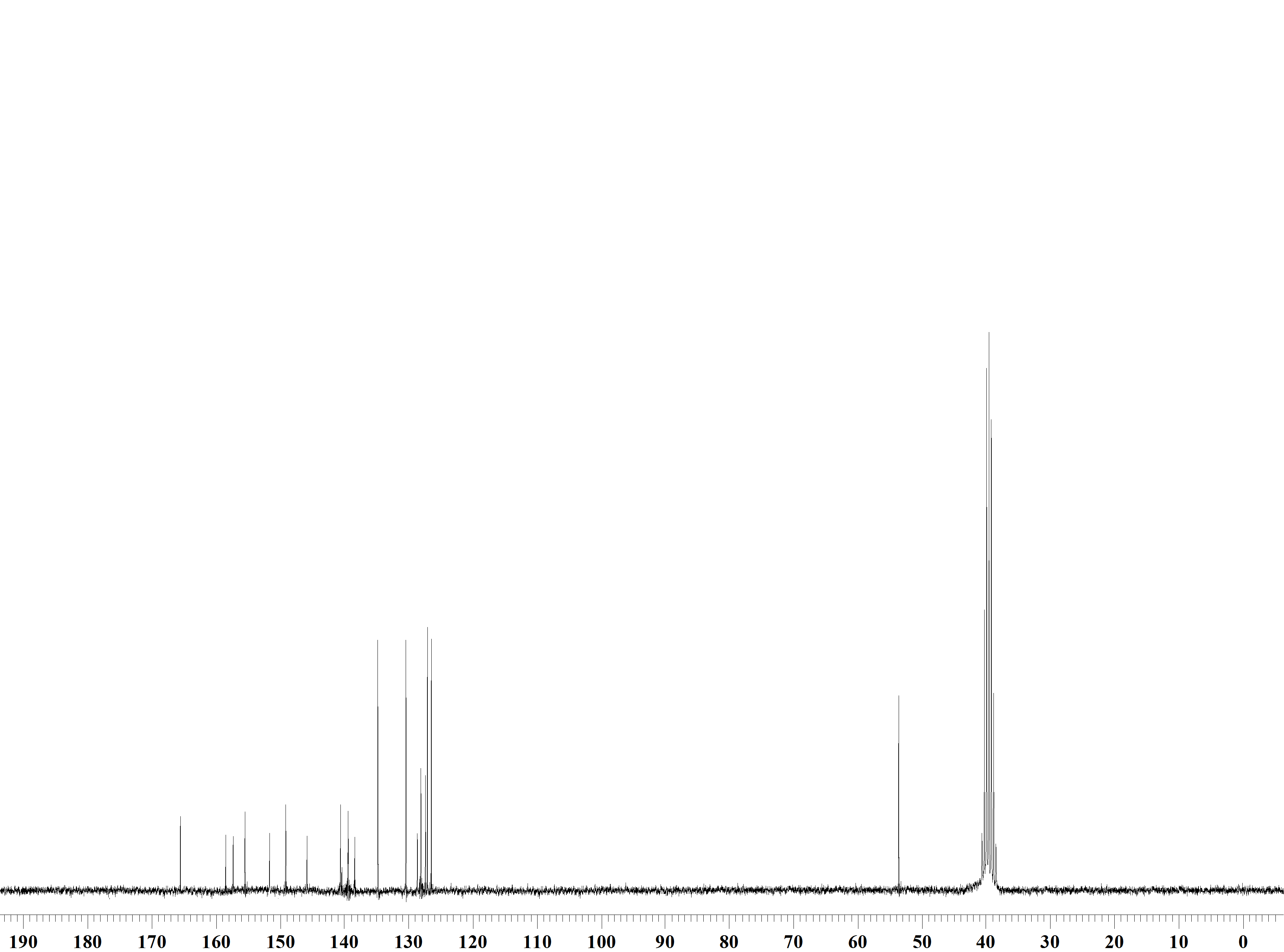


^13^C NMR Spectrum of **7g** (75 MHz, DMSO-d6)


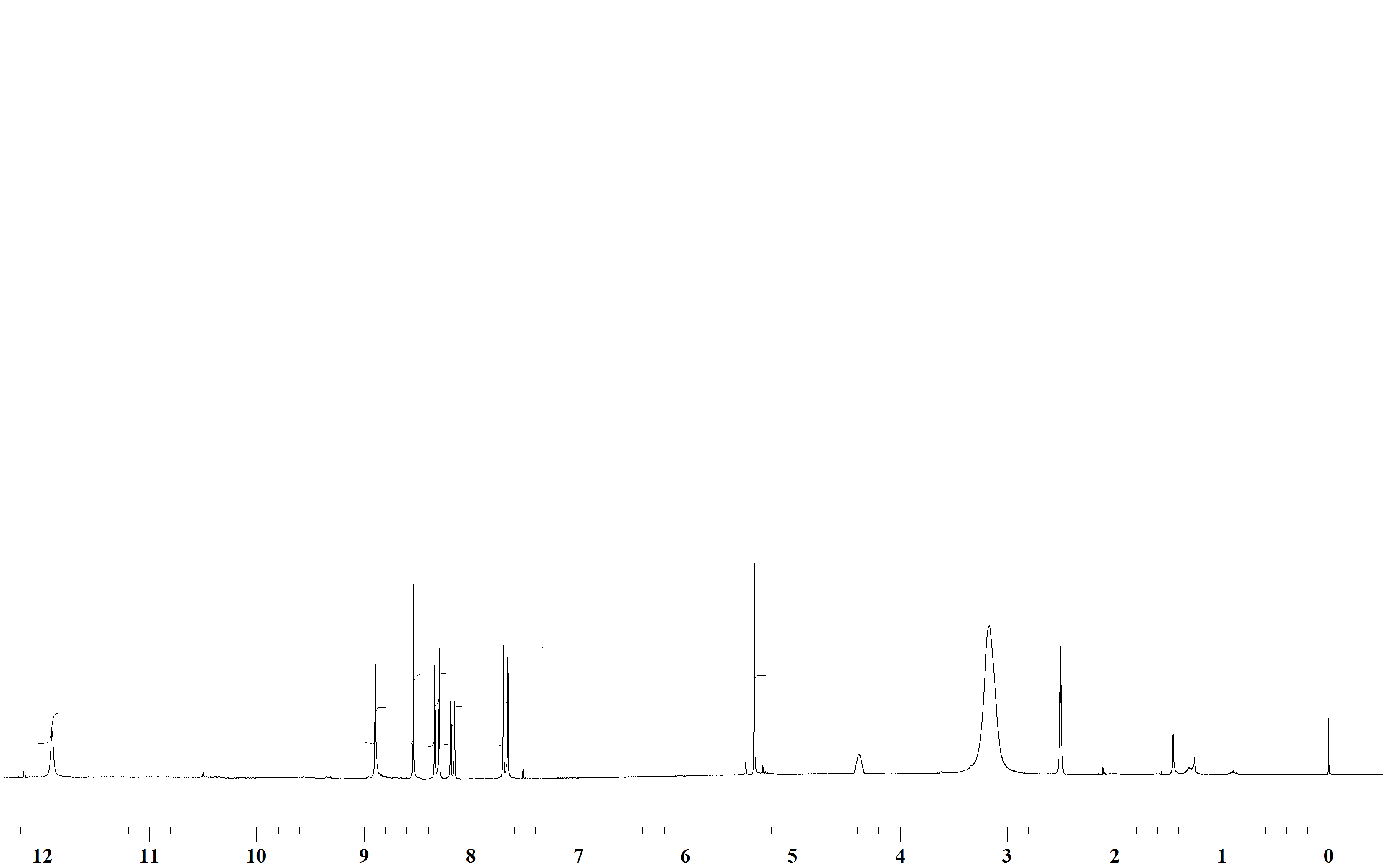


^1^H NMR Spectrum of **7h** (300 MHz, DMSO-d6)


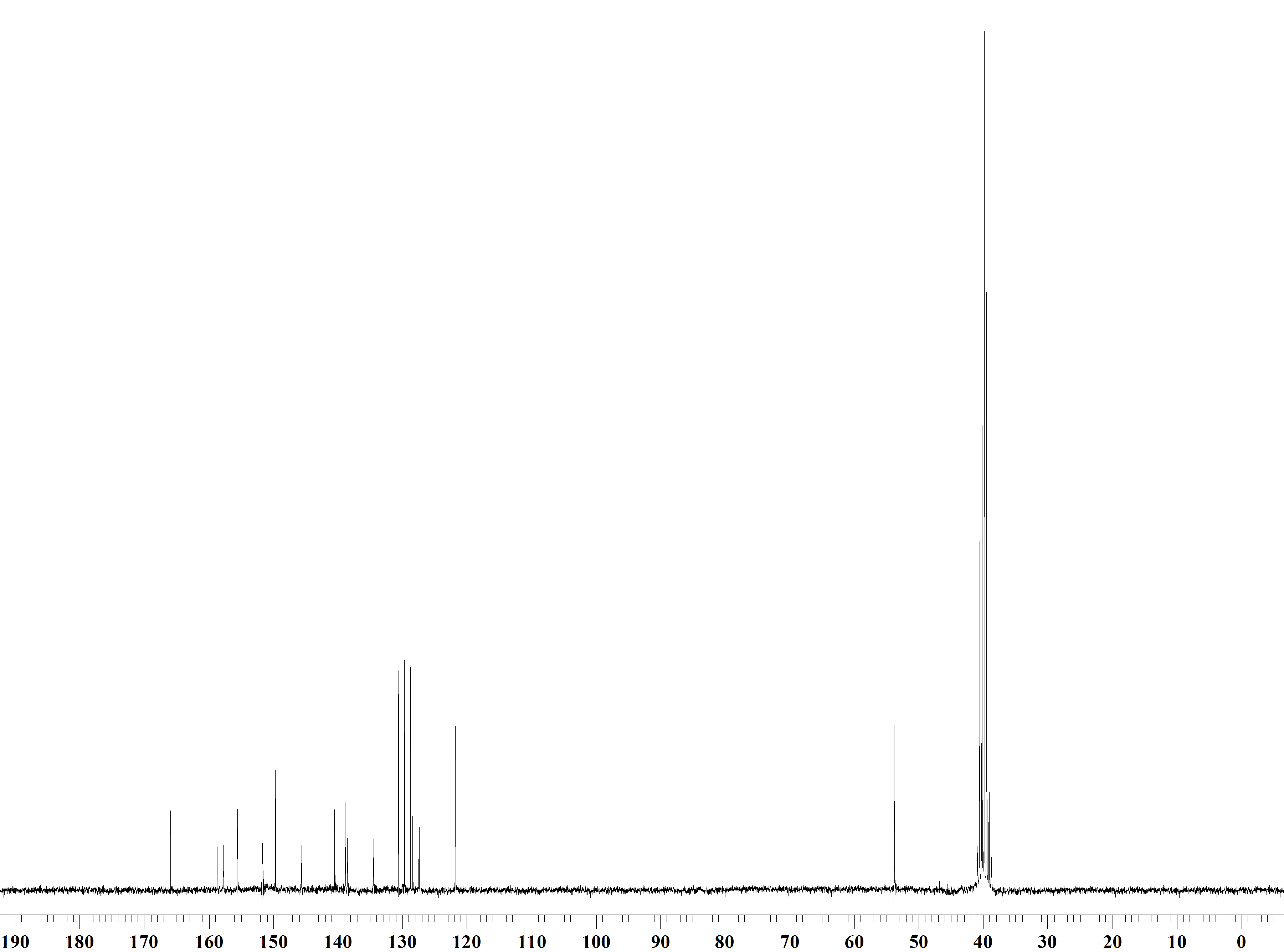


^13^C NMR Spectrum of **7h** (75 MHz, DMSO-d6)


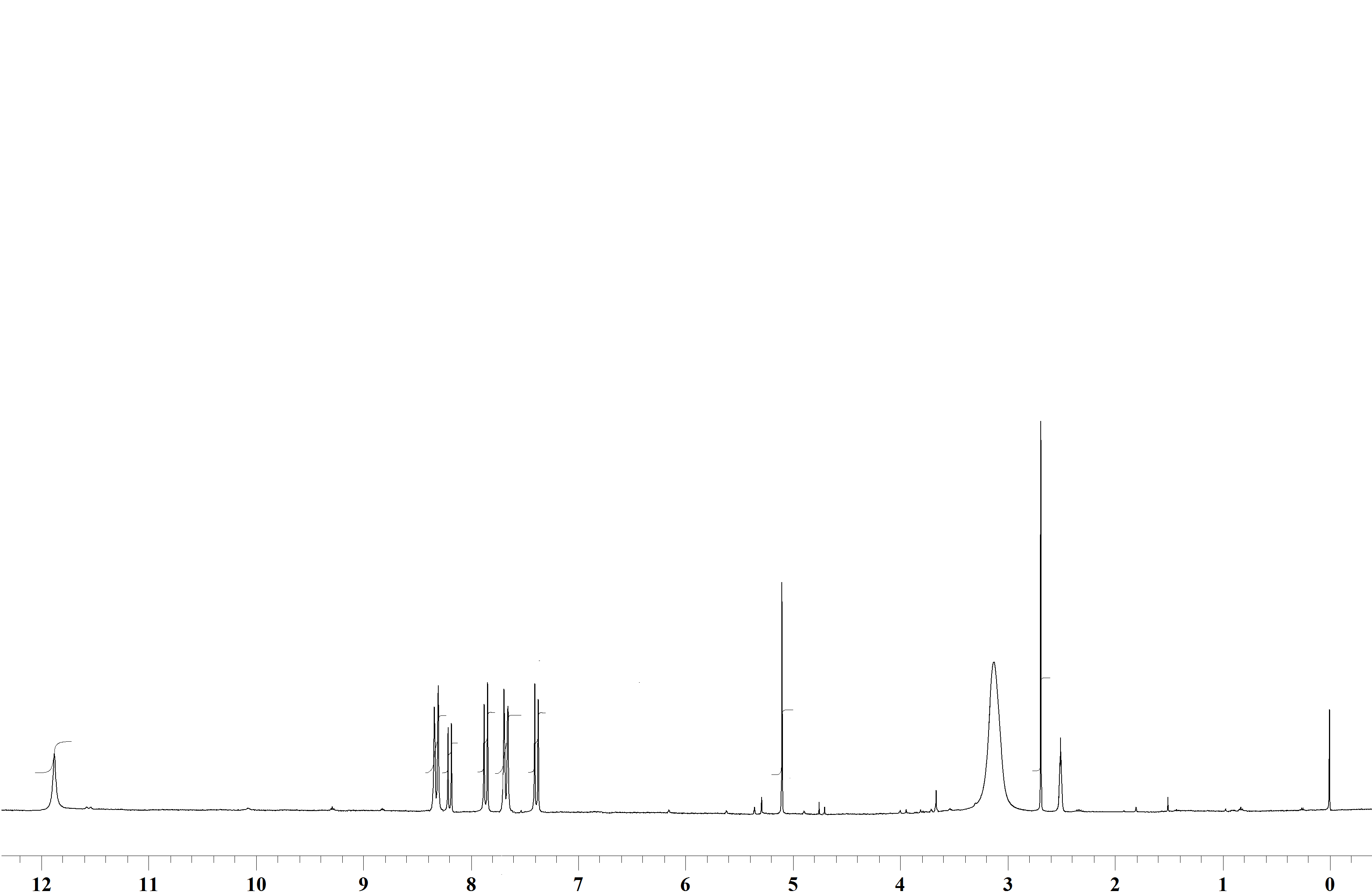


^1^H NMR Spectrum of **7i** (300 MHz, DMSO-d6)


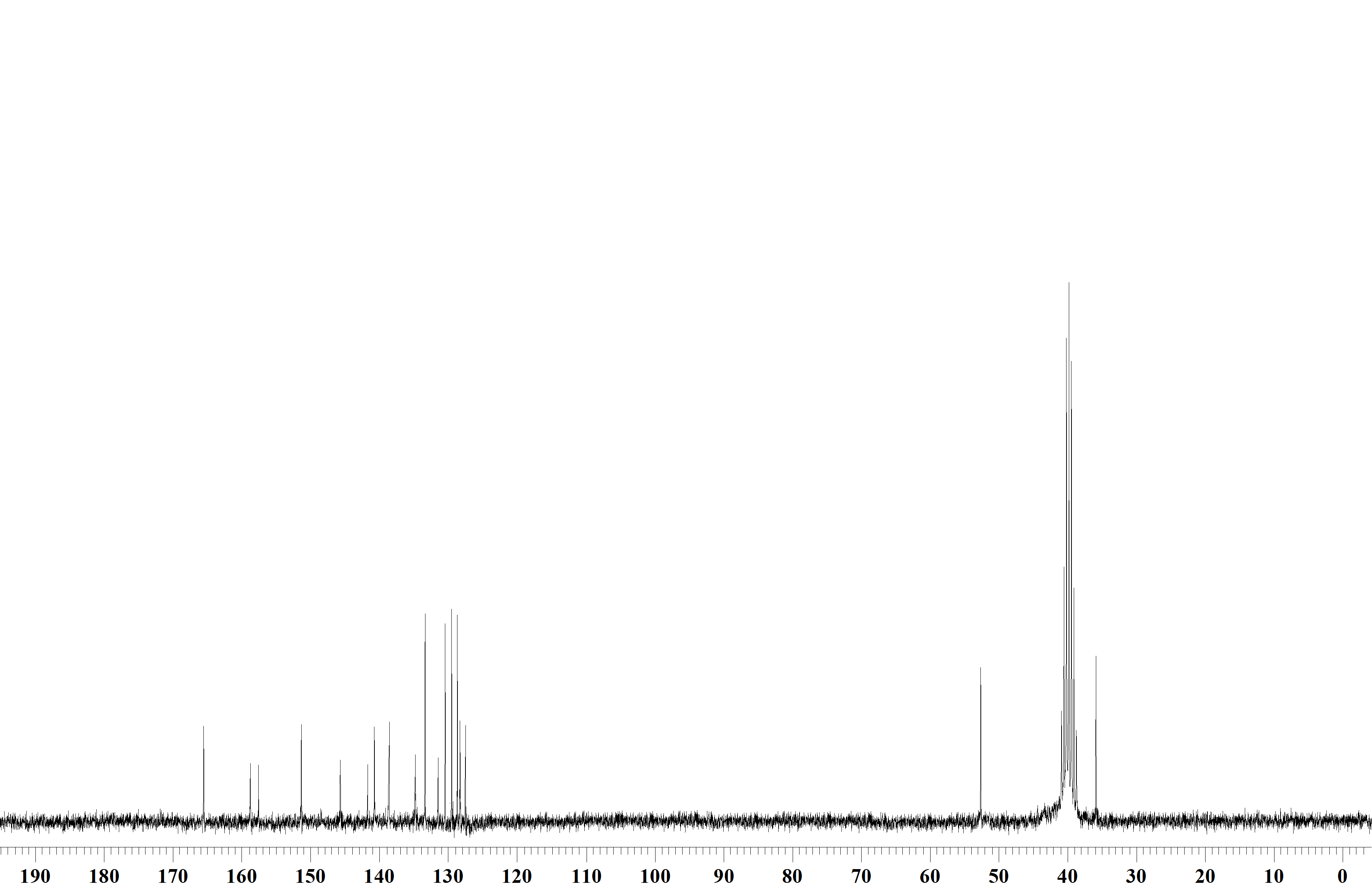


^13^C NMR Spectrum of **7i** (75 MHz, DMSO-d6)


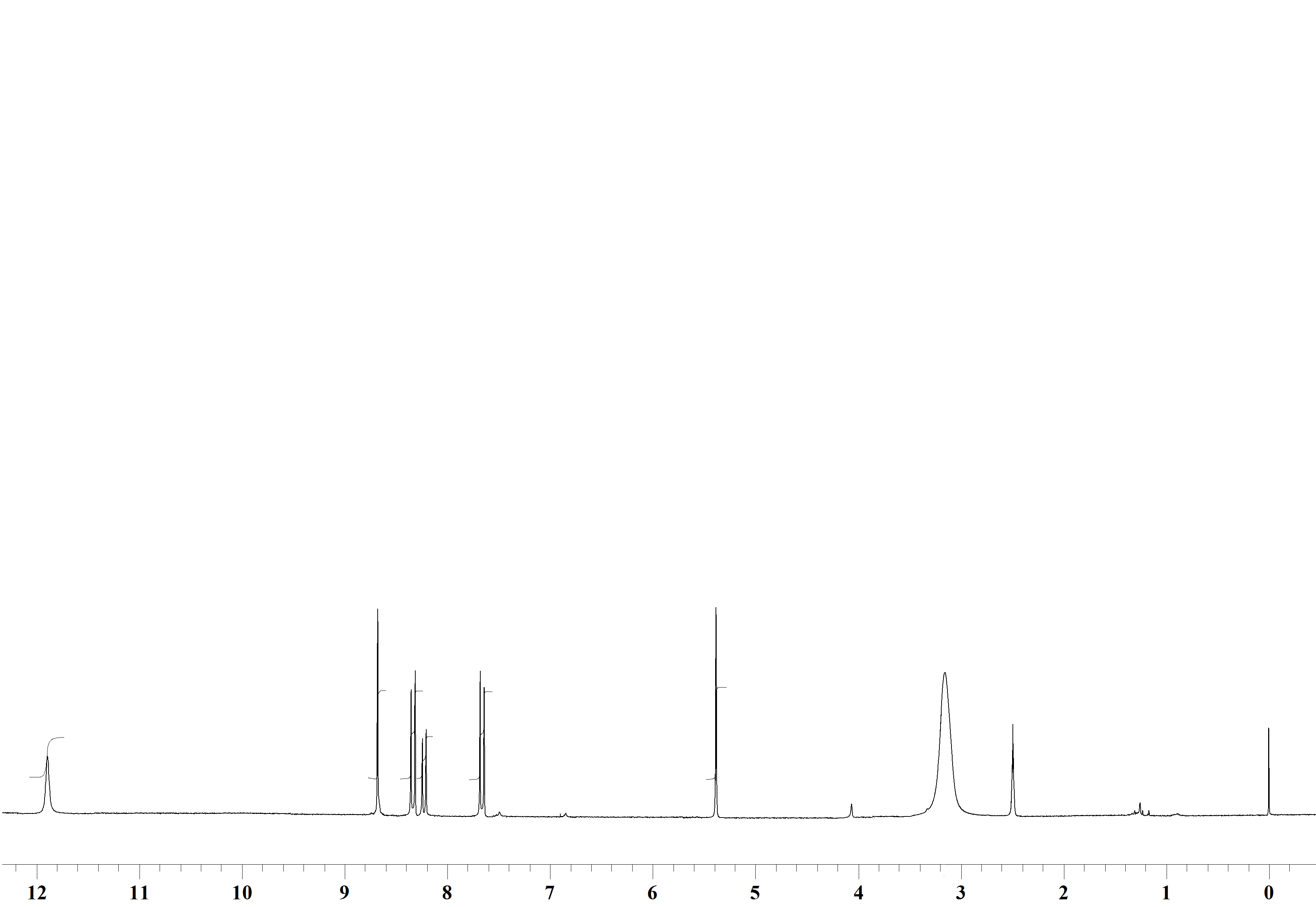


^1^H NMR Spectrum of **7j** (300 MHz, DMSO-d6)


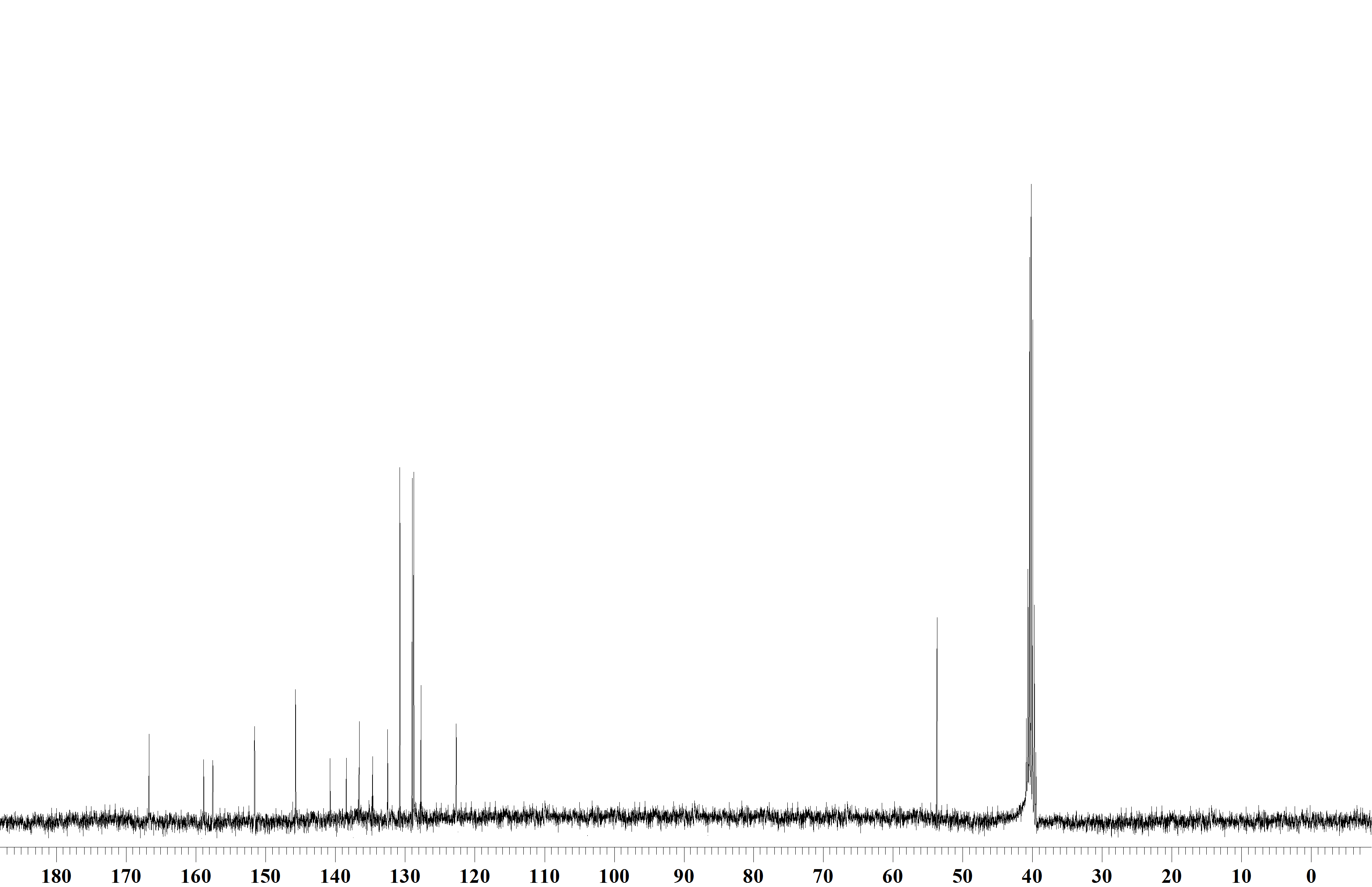


^13^C NMR Spectrum of **7j** (75 MHz, DMSO-d6)
